# The Associations of Maternal Prepregnancy Body Mass Index With Human Milk Fatty Acid and Phospholipid Composition in the Observational Norwegian Human Milk Study

**DOI:** 10.1016/j.tjnut.2025.04.009

**Published:** 2025-04-12

**Authors:** Talat B Ahmed, Merete Eggesbø, Rachel Criswell, Hans Demmelmair, Martina Totzauer, Berthold Koletzko

**Affiliations:** 1 Division of Metabolic and Nutritional Medicine, Department of Paediatrics, Dr. von Hauner Children’s Hospital, LMU University Hospital, Munich, Germany; 2 German Center for Child and Adolescent Health, site Munich, Germany; 3 Department of Paraclinical Sciences, Norwegian University of Life Sciences, Norway; 4 Skowhegan Family Medicine, Redington-Fairview General Hospital, Skowhegan, ME, USA

**Keywords:** human milk, fatty acid, phospholipid composition, prepregnancy body mass index

## Abstract

**Background:**

Human milk fat quality depends on its fatty acid (FA) and phospholipid (PL) composition. There is clear evidence that maternal diet influences human milk FA composition. However, the scientific literature concerning associations between prepregnancy body mass index (pBMI) and milk FA and PL composition remains inconclusive.

**Objectives:**

This observational study aimed to identify the associations between maternal pBMI and the milk FA and choline-containing PL species composition, considering study confounders, including fish intake as a proxy for n3 long-chain polyunsaturated FA (n3-LCPUFA).

**Methods:**

We analyzed total FA and choline-containing PL-classes (lysophosphatidylcholine, phosphatidylcholine, and sphingomyelin) in 628 milk samples from the Norwegian Human Milk Study birth-cohort using gas chromatography and flow-injection mass spectrometry, respectively. Multiple regression analysis assessed the relationship between pBMI and milk lipid metabolites (%FA, %PL) (reported as *β* = standardized regression coefficient with adjusted *P* value < 0.0005, B(95% confidence interval [CI]) = unstandardized coefficient with 95% CI).

**Results:**

Maternal pBMI showed significant association (*P* < 0.0005) with n3-LCPUFA [*β* = −0.138, B(95% CI) = −0.010 (−0.015, −0.005)], n6/n3LCPUFA ratio [*β* = 0.170, B(95% CI) = 0.020(0.012, 0.028)], monounsaturated FA [*β* = 0.207, B(95% CI) = 0.128(0.076, 0.180)], and corresponding PL species [%LysoPC16:1, *β* = 0.171, B(95% CI) = 0.001(0.001,0.002), %LysoPC18:1, *β* = 0.155, B(95%CI) = 0.005 (0.002,0.007)] adjusted with the study covariates. The percentages of variance explained by pBMI were 40% for the n6/n3 LCPUFA ratio, 34% for n3-LCPUFA, and 10% for monounsaturated FA. Conversely, analyses revealed no significant associations between pBMI and choline-containing PL classes.

**Conclusions:**

Biological factors likely increased stearoyl-CoA desaturase activity, lower lipoprotein lipase activity, and a compensatory higher contribution of nonesterified FA from adipose tissue in mothers with pBMI ≥30 could potentially lead to the observed outcomes. Metabolic differences regarding BMI variances may influence the FA availability for mammary gland triglyceride and PL synthesis. Therefore, in addition to dietary intake, maintaining a healthy maternal pBMI may improve the nutritional quality of human milk, ultimately supporting infants’ development.

## Introduction

Human milk is the optimal infant nutrition, delivering nutrients and bioactive components [1,2]. Lipids in human milk play a critical role as an essential energy source. Additionally, they provide structural components, essential fatty acids (FAs), and lipid-soluble vitamins. Human milk fat is primarily composed of triacylglycerol (TAG, 98%), but it also contains phospholipids (PL) and cholesterol [3] with presumed biological importance for the recipient infant. The macro and micronutrient composition of human milk is influenced by maternal, sociodemographic, and infant-related factors, as well as procedures of milk collection and analysis [4,5], with the most profound variation observed for human milk FA composition [3,6].

Maternal diet plays a crucial role in shaping the nutritional quality of human milk [7,8]. For example, increased maternal consumption of n6-polyunsaturated fatty acid (n6-PUFA), compared to n3-PUFA, is reflected in human milk FA and may affect developmental outcomes [9]. Multiple studies suggested that maternal prepregnancy obesity or gestational weight gain is linked to the ratio of unsaturated to saturated FA in human milk [[10], [11], [12]]. Notably, mothers with obesity tend to have a higher n6 to n3-FA ratio compared with normal-weight mothers. This imbalance is often attributed to differences in the FA composition of dietary fats despite no significant changes in total fat intake [[9], [10], [11], [12], [13]]. However, Ellsworth et al. [14] found no alterations in the total n6 to n3-long-chain PUFA (LCPUFA) ratio in milk of mothers with obesity, although they reported increased percentages of individual FA, such as palmitic acid (C16:0), dihomo-γ-linolenic acid (C20:3n–6), and adrenic acid (C22:4n–6) [14]. Recently, Hua et al. [15] identified nonsignificant differences in saturated FA, MUFA, n3, n6-PUFA, and n6/n3 PUFA ratio between the mature milk of lean and mothers with obesity [15]. Thus, despite numerous studies, it remains unclear whether obesity systematically affects the FA composition of human milk.

Human milk PLs, mainly phosphatidylcholine (PC), phosphatidylethanolamine, and sphingomyelin (SM) [16], play a critical role in TAG transport and contain bioactive components, including choline and LCPUFA, which are essential for maintaining the optimal nutritional status of infants [17,18]. Despite their importance, the influence of maternal weight status on milk PL composition remains to be determined. Serum-based studies among young adults found significant associations between obesity and the choline-containing PL subclasses (LysoPC, PC, and SM), suggesting that obesity and inflammation may be associated with altered PL concentrations [[19], [20], [21]].

Obesity is linked with low-grade inflammation, which includes the release of tumor necrosis factor-α (TNF-α) from macrophages and adipocytes. This triggers sphingomyelinase activation and elevates ceramide levels [22]. Additionally, obesity can disrupt ceramide metabolism due to the increased availability of plasma-free FAs, proinflammatory cytokines, and oxidative stress [23]. Available FAs impact sphingolipid levels through both substrate supply and regulation of the enzymes involved in sphingolipid metabolism [23,24]. Moreover, SMs are synthesized from ceramides and PCs by SM synthase activity [24], suggesting that SM and PC molecular species might be altered in mothers with obesity. These findings support the hypothesis that maternal prepregnancy body mass index (pBMI) may be associated with the composition of FA and choline-containing PL in milk. Our study aimed to examine associations between maternal pBMI, human milk FA, and choline-containing PL species composition in a subset of Norwegian mothers’ milk samples obtained from the Norwegian Human Milk Study (HUMIS) birth cohort.

## Methods

### Study design and population

**FIGURE 1:**
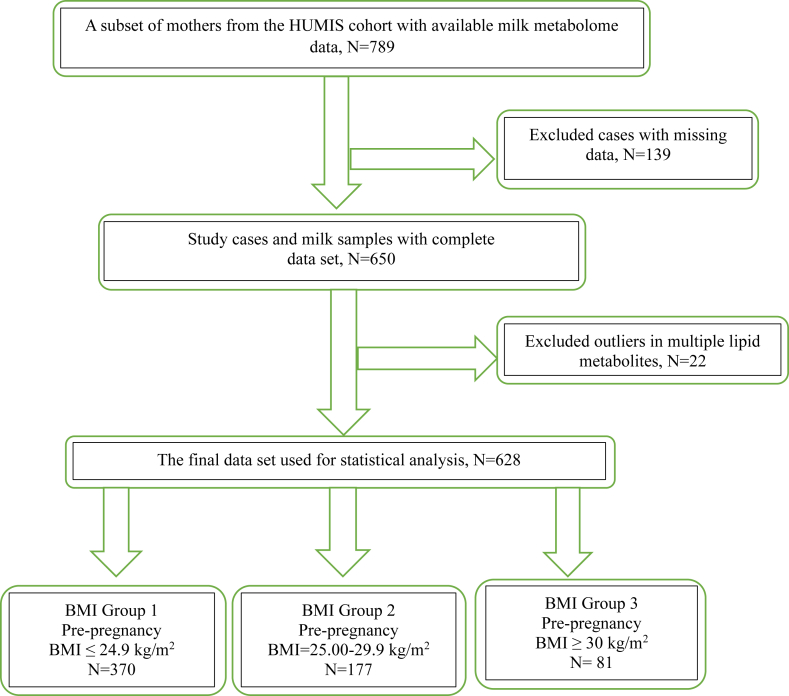
Flow diagram depicting the selection process of the subjects for the present analysis. BMI stands for body mass index in kg/m^2^.

In this observational study, we utilized human milk samples from the HUMIS birth cohort, which included 2606 women recruited from 2002 to 2009 across Norway. A subset of this cohort (*n* = 789), oversampled for maternal overweight and obesity status, was selected for this study. The milk sampling process has previously been described in detail [25,26]. Briefly, mothers were asked to collect and freeze hand-expressed or pumped 25 mL samples of their first milk before feeding their infants each morning for 8 consecutive days, starting not earlier than 2 wk postpartum. Milk collection ranged from 14 to 108 d postpartum, with a median collection time at day 31 postpartum. After collection, the samples were pooled, transferred to the Norwegian Institute of Public Health in Oslo, and stored at −20°C. Additionally, mothers completed questionnaires regarding their weight and other relevant factors. We excluded 139 mothers for the present study due to missing data in multiple variables, including study exposure (maternal pBMI), resulting in a sample of 650 women (Figure 1). Written informed consent was obtained from all participants before enrollment. The study was approved by the Norwegian Data Inspectorate and the Norwegian Regional Committee for Medical and Health Research Ethics.

### Measurements

The milk samples (5 mL) were shipped on dry ice to Hauner Children’s Hospital in Munich for analysis. Laboratory analyses have been described elsewhere [27,28]. Briefly, the milk samples were thoroughly mixed and thawed in a water bath at 20°C to 30°C. To analyze total FA and PL species, 100 μL of milk was combined with 700 μL of methanol and 600 μL of methyl tert-butyl ether (contained triundecanoin as an internal standard), then vortexed for 30 s and centrifuged without temperature control at 1500 × *g* for 5 min to precipitate proteins. In an aliquot of the supernatant, total FA was transferred into their methyl esters and analyzed using gas chromatography (Agilent 7890 GC with Gerstel KAS 4 injector). The FA methyl esters were separated, identified, and quantified (50 m BPX-70 column, SGE, and GLC569 Nu-Chek-Prep Inc) by using the previously established method [29]. Thirty-two FAs with chain lengths ranging from C8:0 to C22:0 were quantified as absolute concentrations (g/L) and presented as weight/weight percentages (wt/wt%), including n6/n3 PUFA and LCPUFA utilized as ratios in statistical analysis. The mean coefficient of variation (%CV) was found to be 13% for concentrations and 9% for percentages based on quality control (randomly distributed 82 aliquots of a reference milk sample).

To analyze PL metabolites, a 100-μL aliquot of the supernatant was combined with 500 μL of methanol containing 0.4 g/L of ammonium acetate and internal standards (lyso-PC13:0, PCaa.C28:0) used for flow-injection tandem mass spectrometry (Agilent, Waldbronn, 4000QTRAP, Sciex) as previously described [19,27]. The measurements were carried out using Analyst 1.5.1 software (Sciex), following previously established protocols for plasma samples [19,30]. We quantified 330 molecular species that exhibited highly variable precision using this method. We selected PL metabolites for this study based on their high abundance and included only those lipid metabolites (161 metabolites) whose average contribution exceeded 0.01 mol%. Additionally, we analyzed 66 aliquots of a milk sample as quality control alongside the study samples in each batch; ultimately, the final selection of metabolites (67 metabolites) was based on their relative composition > 0.01%, with %CV below 35%. The analysis comprised lysophosphatidylcholine (lyso-PC), Phosphatidylcholine (PC), including diacyl-phosphatidylcholine (PCaa), acyl-alkyl phosphatidylcholine(PCae), and sphingomyelin (SM). The flow-injection mass spectrometry technique does not provide information about the position of double bonds or the distribution of carbon atoms between FA. To address this limitation, we adopted the XX: Y nomenclature for PL species, where XX indicates the carbon chain length, and Y represents the number of double bonds. Furthermore, if an acyl chain is esterified via an ester bond, we use “aa,” whereas “ae” denotes the presence of an ether bond.

### Data treatment and statistical analysis

We used maternal pBMI (kg/m²) as a continuous and categorical independent variable as the study exposure. The categorization of BMI followed the guidelines set by the WHO: underweight + normal weight (UW+NW) ≤ 24.9, overweight (OW) = 25.0–29.9, and obese (OB) ≥ 30 [31].

Lipid metabolites, 32 FAs (percentage of total FA), and 67 PL (percentage of total PL) species were included as study outcome variables. The normal distribution of variables was evaluated using the Shapiro–Wilk test and by visually inspecting Q-Q plots. We used box-whisker plots to identify outliers. We considered any value outside the range of Q1 - (1.5 IQR) and Q3 + (1.5 IQR) as an outlier [32] and found 18 outliers across multiple lipid metabolites. For multivariate outliers, we conducted a Mahalanobis distance test [33], which identified 4 outliers that were subsequently removed to obtain a final sample size of 628 subjects (Figure 1).

We identified study covariates (maternal age, diet, education, parity, and smoking status), mediators such as gestational weight gain and mode of delivery, and ancestors of outcomes such as gestational age and lactational stage from the literature [5,8,[34], [35], [36]], and collected relevant information from mothers’ completed questionnaires. Additional data from the Norwegian Medical Birth Registry and the mothers' medical pregnancy journals were also utilized to add the information related to study covariates (maternal smoking, education, parity).

We accounted for the consumption of fatty fish in the previous year, defined as having ≥2 dinners per week that included fatty fish (with 7 bread meals, an open-faced sandwich with fatty fish counted as equivalent to one dinner). We also considered cod liver oil consumption over the course of a year to be a significant potential confounder for our study. Data on maternal diet was collected through a questionnaire administered to mothers at a median age of 1 mo after delivery. The section on diet was titled “Your Diet Over the Last Year.” Mothers were asked to report their intake frequency—either weekly, monthly, or yearly—for specifically defined food items and groups. These included cod liver oil, mackerel, salmon, trout, herring, lean fish, halibut, pike, perch, and processed fish.

The categories used in the questionnaire were similar to those in the Food Frequency Questionnaire utilized in the Norwegian MoBa study, which has been validated [37]. To determine the minimally sufficient adjustment set, we used a directed acyclic graph (DAG) through DAGgitty v3.0 [38] (Supplemental Figure 1). The final regression model included the following covariates: maternal age (y), fish intake and cod liver oil consumption (continuous, servings in d/y), education (<12 or ≥12 years), parity (primiparous versus multiparous), gestational weight gain (continuous, kg), delivery mode (vaginal versus cesarean), and smoking status (never smoking versus ever smoking).

Maternal characteristics and continuous variables are expressed as mean ± standard deviations. ANOVA and Bonferroni post-hoc tests were employed to explore the differences in maternal demographic characteristics and milk lipid metabolites between different pBMI groups; the chi-square test was used for categorical variables. Both unadjusted and adjusted linear regression models were employed. Associations are reported as β = standardized regression coefficient with adjusted *P* value < 0.0005, B(95% CI) = unstandardized coefficient with 95% confidence interval (95% CI). The statistical significance level was set to *P* value < 0.05, and a Bonferroni-adjusted *P* value of 0.0005 (=0.05/99) was used for multiple comparisons. All statistical analyses were performed using the SPSS statistical software package for Windows (version 29; IBM SPSS, Ink). We conducted the linear regression analysis with (*n* = 650) and without (*n* = 628) outliers to observe their influence on associations. As the constant-sum constraint may impact the outcomes of percentage data analysis [39], a subset of milk PL data from the cohort was transformed using the centered log ratio (CLR) method, and corresponding associations were tested.

## Results

In our study subset, 59% of mothers were classified as non-obese, whereas 28% were classified as overweight and 13% as obese (Supplemental Table 1). We observed significant differences in maternal gestational weight gain and the percentage of cesarean delivery between the normal weight and the other BMI groups, although there were no significant differences in fish intake (proxy for diet rich in n3-LCPUFA), smoking status, education, parity, and gestational age of infants among pBMI groups (Table 1). The percentage distribution of individual and grouped FA in mothers' milk among pBMI groups is presented in Table 2. Milk from mothers with higher pBMI (pBMI > 24.9) demonstrated a trend toward higher percentages of saturated FA and MUFA (*P* < 0.05) and lower percentages of odd chain FAs (*P* < 0.0005), additionally displayed significantly different levels of n3-LCPUFA, C22:4n–6, and n6/n3-LCPUFA ratio compared with milk from mothers with normal weight. Choline-containing PL percentages in mothers' milk varied and displayed inconsistent differences across pBMI groups, and no significant differences in choline-containing PL classes and individual species composition were observed between the groups (Supplemental Table 2).

**TABLE 1:** The comparison of characteristics of the study population in a subset (*n* = 628) according to maternal prepregnancy body mass index.

Maternal characteristics	Total (*n* = 628)	Normal weight (*n* = 370)	Overweight (*n* = 177)	Obese (*n* = 81)	*P* value
Age (y)	29.49(4.26)	29.22(4.45)	29.93(4.00)	29.74(4.18)	0.165
Prepregnancy weight (kg)	69.67(13.23)	61.72(6.88) ^a^	75.32(6.08) ^b^	93.63(11.35) ^c^	<0.001∗
Prepregnancy BMI (kg/m² )^1^	24.84(4.57)	21.85(1.92) ^a^	27.05(1.40) ^b^	33.70(3.30) ^c^	<0.001∗
Weight at delivery (kg)	84.89(13.50)	77.60(9.18) ^a^	91.14(8.60) ^b^	104.53(12.47) ^c^	<0.001∗
Gestational weight gain (kg)^2^	15.22(5.85)	15.89(5.40) ^a^	15.82(5.83) ^a^	10.90(6.30) ^b^	<0.001∗
Excess gestational weight gain (%)^3^					<0.001∗
Yes	58.80	48.60 ^b^	79.10 ^a^	60.50 ^ab^	
Mode of delivery (%)					
Vaginal delivery	84.20	88.60 ^a^	81.40 ^ab^	70.40 ^b^	
Cesarean section	15.80	11.40 ^a^	18.60 ^ab^	29.60 ^b^	
Parity (%)					
Primiparous	39.60	41.40	40.70	29.60	0.141
Multiparous	60.40	58.60	59.30	70.40	
Maternal education (%)					
<12-y education	8.30	8.10	6.20	13.60	
≥12-y education	91.70	91.90	93.80	86.40	0.135
Smoking status (%)					
Nonsmokers	58.40	59.20	60.50	50.60	
Past + current smoker	41.60	40.80	39.50	49.40	0.298
Dietary n3-LCPUFA-rich diet Intake (servings in d/y)					
Cod liver oil consumption	147.00(163.18)	161.88(162.49)	134.95(166.51)	105.35(150.56)	0.009
Lean fish (cod) dinner	33.56(31.33)	33.93(31.63)	33.98(31.96)	30.93(28.66)	0.723
Vegetarian dinner	34.40(39.49)	37.11(42.90)	34.08(35.68)	26.85(28.94)	0.100
Fatty fish dinner	25.83(28.42)	27.60(30.81)	22.90(24.25)	24.14(24.88)	0.166
Infant Characteristics					
Gestational age (days)	280.20(12.78)	279.76(12.85)	281.53(11.60)	279.20(14.68)	0.247
Birth weight (gm)	3638.0(592.5)	3544.7(556.2) ^a^	3776.0(577.6) ^b^	3763.4(702.2)^b^	<0.001∗
Infant Sex					0.743
Boy		52.40	53.10	51.90	
Girl		47.60	46.90	48.10	

Abbreviation: BMI: Body mass index.

Data expressed as mean ± SD for continuous variables and *n* (%) for categorical variables.

1 BMI groups: normal weight (NW) ≤ 24.9, overweight (OW) = 25.0–29.9, and obese (OB) ≥ 30.

2 Defined as the difference in weight gain in kilograms between maternal weight at delivery and prepregnancy.

3 Defined per body mass index category according to Institute of Medicine guidelines: for underweight women, >18.1 kg; for normal weight women, >15.9 kg; for overweight women, >11.3 kg; and for obese women, >9.1 kg. According to ANOVA and the Bonferroni post-hoc test, different superscript letters indicate differences among BMI groups. The chi-square test was applied to qualitative variables. Means in a row not sharing superscripts are significantly different from one another.

**Table 2:** Fatty Acid profile in human milk samples (n=628) according to maternal pre-pregnancy BMI.

%FA	Total Study Samples (n=628) Mean (SD)	Normal-weight (n=370) Mean (SD)	Overweight (n=177) Mean (SD)	Obese (n=81) Mean (SD)	p-value
1SFA C8:0 C10:0 C12:0 C14:0 2MCFA C13:0 C15:0 C17:0 3ODCFA	47.11 (3.56) 0.34 (0.093) 1.64 (0.35) 5.94 (1.61) 6.02 (1.38) 13.94 (3.21) 0.11 (0.04) 0.36 (0.09) 0.30 (0.04) 0.78 (0.14)	47.26 (3.37) 0.35 (0.092) 1.66 (0.36) 6.02 (1.62) 6.09 (1.37) 14.13 (3.20) 0.11 (0.05) 0.37 (0.10)^a^ 0.31 (0.04) 0.79 (0.15)^a^	46.56 (3.71) 0.34 (0.093) 1.57 (0.34) 5.70 (1.57) 5.80 (1.50) 13.34 (3.13) 0.11 (0.04) 0.35 (0.08)^ab^ 0.31 (0.04) 0.77 (0.13)^a^	47.63 (3.93) 0.34 (0.10) 1.66 (0.35) 6.15 (1.60) 6.22 (1.38) 14.37 (3.31) 0.10 (0.04) 0.33 (0.08)^b^ 0.30 (0.04) 0.72 (0.13)^b^	0.034 0.329 0.021 0.023 0.012 0.011 0.054 <0.001 0.012 <0.001*
C16:0 C18:0 C22:0 4LCSFA 5MUFA C14:1 C15:1	23.06 (1.67) 9.18 (1.24) 0.16 (0.05) 32.40 (2.23) 36.65 (2.82) 0.25 (0.06) 0.08 (0.02)	22.91 (1.60) 9.27 (1.21) 0.16 (0.05) 32.34 (2.20) 36.34 (2.80) 0.25 (0.07) 0.08 (0.02)	23.26 (1.76) 9.03 (1.26) 0.15 (0.04) 32.44 (2.27) 37.12 (2.84) 0.26 (0.06) 0.08 (0.02)	23.31 (1.80) 9.07 (1.24) 0.16 (0.04) 32.54 (2.24) 37.00 (2.86) 0.24 (0.06) 0.07 (0.02)	0.024 0.068 0.252 0.725 0.005 0.187 0.132
C16:1n-7	2.50 (0.62)	2.40 (0.57)^a^	2.60 (0.70)^b^	2.50 (0.63)^ab^	<0.001
C18:1n-7 C18:1n-9 C20:1n9 6tFA C16:1t C18:1t C22:1t 7PUFA C20:3n-9 N6-PUFA C18:2n-6 C18:3n-6 C20:2n-6 C20:3n-6 C20:4n-6 C22:4n-6 C22:5n-6 N3-PUFA C18:3n-3 C20:3n-3 C20:5n-3 C22:5n-3 C22:6n-3 8LCPUFA N6-LCPUFA N3-LCPUFA 9N6/N3-PUFA 10N6/N3-LCPUFA 11Un-SFA/SFA Total FA (g/l)	1.63 (0.22) 31.80 (2.50) 0.46 (0.10) 0.62 (0.19) 0.09 (0.02) 0.45 (0.18) 0.09 (0.03) 15.61 (2.85) 0.020 (0.01) 13.81 (2.62) 12.42 (2.53) 0.127 (0.04) 0.465 (0.14) 0.350 (0.08) 0.360 (0.07) 0.065 (0.02) 0.025 (0.01) 1.80 (0.47) 1.07 (0.29) 0.047 (0.01) 0.102 (0.07) 0.165 (0.05) 0.393 (0.21) 1.53 (0.35) 0.80 (0.14) 0.70 (0.32) 8.14 (1.90) 1.33 (0.54) 1.12 (0.16) 24.08 (8.70)	1.60 (0.21) 31.55 (2.50) 0.47 (0.11) 0.64 (0.20) 0.09 (0.03) 0.46 (0.19) 0.10 (0.03) 15.75 (2.79) 0.02 (0.01) 13.90 (2.60) 12.51 (2.48) 0.126 (0.04) 0.473 (0.15) 0.347 (0.08) 0.357 (0.07) 0.062 (0.02)^a^ 0.025 (0.01) 1.83 (0.50)^a^ 1.078 (0.31) 0.048 (0.01) 0.110 (0.07)^a^ 0.171 (0.06)^a^ 0.422 (0.21)^a^ 1.56 (0.37)^a^ 0.792 (0.14) 0.751 (0.34)^a^ 7.98 (1.97)^a^ 1.25 (0.53)^a^ 1.11 (0.15) 23.62 (8.53)	1.68 (0.23) 32.05 (2.41) 0.45 (0.09) 0.62 (0.17) 0.09 (0.02) 0.45 (0.16) 0.09 (0.03) 15.70 (2.80) 0.02 (0.01) 13.90 (2.59) 12.49 (2.51) 0.133 (0.04) 0.456 (0.13) 0.356 (0.08) 0.370 (0.06) 0.070 (0.03)^b^ 0.026 (0.01) 1.78 (0.45)^a^ 1.073 (0.27) 0.047 (0.01) 0.100 (0.07)^a^ 0.166 (0.06)^a^ 0.385 (0.21)^a^ 1.54 (0.34)^a^ 0.819 (0.13) 0.699 (0.32)^a^ 8.15 (1.80)^a^ 1.37 (0.52)^a^ 1.14 (0.17) 24.71 (8.84)	1.64 (0.26) 32.16 (2.43) 0.42 (0.07) 0.56 (0.17) 0.08 (0.02) 0.40 (0.16) 0.08 (0.02) 14.80 (3.10) 0.02 (0.01) 13.25 (2.85) 11.87 (2.74) 0.122 (0.03) 0.449 (0.15) 0.357 (0.07) 0.352 (0.08) 0.070 (0.02)^b^ 0.025 (0.01) 1.52 (0.34)^b^ 0.990 (0.27) 0.046 (0.01) 0.07 (0.04)^b^ 0.143 (0.05)^b^ 0.280 (0.14)^b^ 1.35 (0.33)^b^ 0.803 (0.14) 0.530 (0.19)^b^ 8.87 (1.58)^b^ 1.64 (0.49)^b^ 1.10 (0.17) 24.82 (9.13)	0.002 0.027 0.002 0.006 0.314 0.032 0.003 0.020 0.677 0.114 0.113 0.069 0.238 0.339 0.093 <0.001* 0.871 <0.001* 0.037 0.114 <0.001* <0.001* <0.001* <0.001* 0.096 <0.001* <0.001 <0.001* 0.025 0.280

%FA - Percentages of individual and grouped FA, total FA (g/L) are presented as mean (SD).

P-values are derived from one-way ANOVA and the Bonferroni post-hoc test. P-values are derived from one-way ANOVA and the Bonferroni post-hoc test. According to ANOVA and the Bonferroni post-hoc test, different superscript letters indicate differences among BMI groups. Means in a row not sharing superscripts are significantly different from one another. P values < 0.05 are reported here; the adjusted level of statistical significance is α = 0.05/99 = 0.0005. P values below the adjusted threshold are marked with a star.

1 Saturated fatty acid,

2 Medium-chain saturated fatty acid,

3 Odd-chain fatty acid,

4 Long-chain saturated fatty acid,

5 Monounsaturated fatty acid,

6 Trans fatty acid,

7 Polyunsaturated fatty acid,

8 Long-chain polyunsaturated fatty acid.

9 N6/N3-PUFA,

10 N6/N3-LCPUFA,

11 Un-SFA/SFA-unsaturated fatty acid/saturated fatty acid as FA ratio.

**FIGURE 2:**
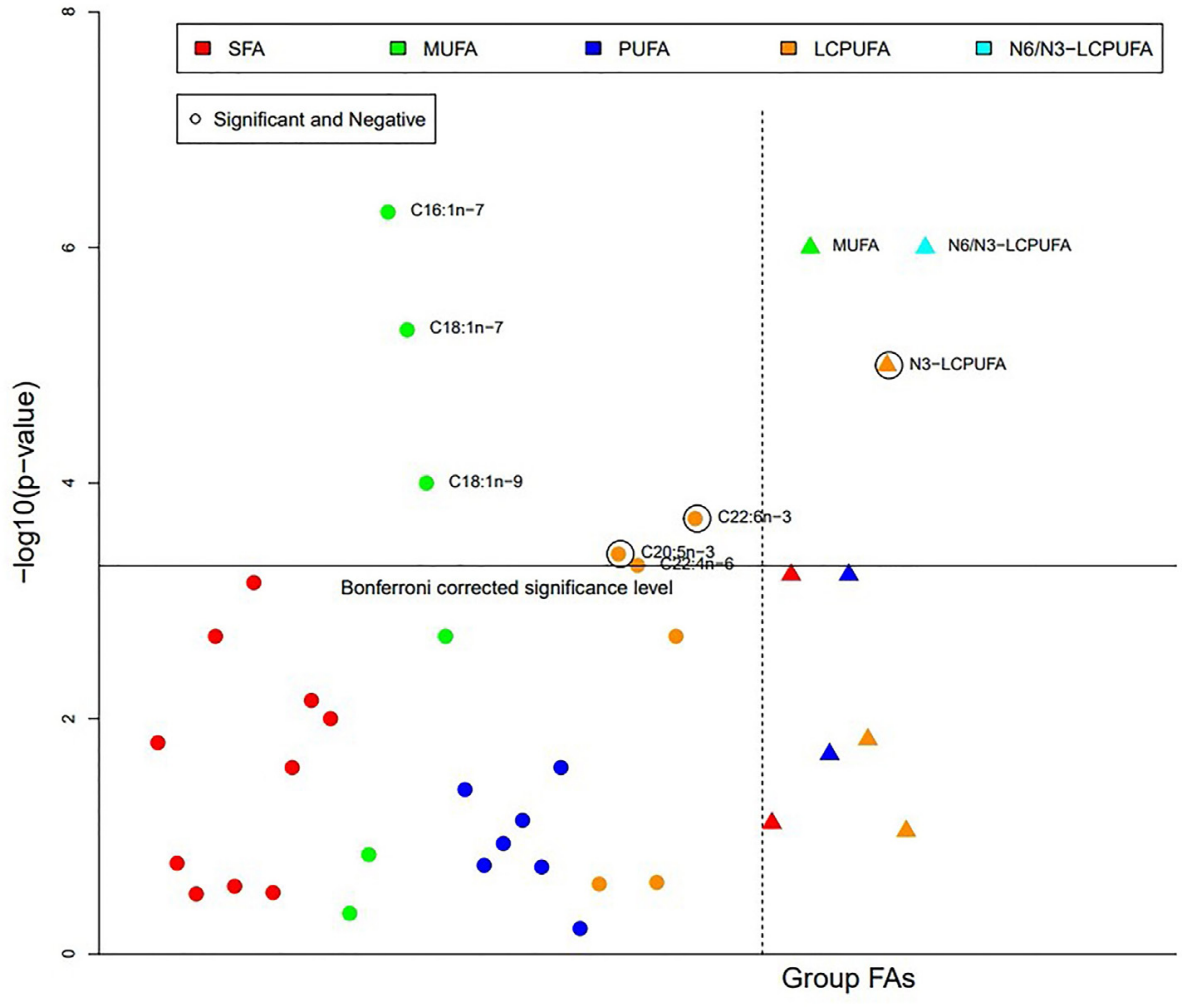
Adjusted associations of milk percentage fatty acid (%FA) with maternal prepregnancy body mass index (pBMI kg/m²). *P* values of the multiple regression models relating maternal prepregnancy BMI kg/m² to milk %FA. The dark horizontal line corresponds to the Bonferroni-corrected significance level of α =0.05/99 (number of analytes). Points are −log10 (*P* values) of the regression model. The independent variable is maternal prepregnancy BMI kg/m²; the dependent variable is the respective %FA. Only significant %FA species are presented with a formula or name, and significantly negatively associated %FAs are encircled. The dotted line separates the individual %FA from the grouped %FA.

Maternal pBMI was significantly positively associated only with MUFA (β = 0.207, B(95%CI) = 0.128(0.076,0.180), *P* < 0.0005) and n6/n3-LCPUFA [β = 0.170, B(95%CI) = 0.020(0.012,0.028), *P* < 0.0005] and negatively associated with n3-LCPUFA [β = −0.138, B(95% CI) = −0.010(−0.015, −0.005), *P* < 0.0005] after adjustment for study covariates. Furthermore, pBMI was positively associated with palmitoleic acid (C16:1n–7) [β = 0.212, B(95% CI) = 0.029(0.018, 0.040), *P* < 0.0005], cis-vaccenic acid (C18:1n–7) [β = 0.195, B(95% CI) = 0.009(0.005, 0.014), *P* < 0.0005], and oleic acid (C18:1n–9) [β = 0.171, B(95% CI) = 0.092(0.046, 0.138), *P* < 0.0005] only in adjusted models (Figure 2, Supplemental Table 3).

Maternal pBMI was also significantly associated with multiple individual LCPUFA in both adjusted and unadjusted analyses (Figure 2, Supplemental Tables 3 and 4). The relationship between pBMI and n6/n3-LCPUFA is one of the closest, explaining almost 40% of the n6/n3-LCPUFA variance after adjustment. In all significant associations, pBMI accounted for >10% of the variance (Supplemental Table 3). Furthermore, different categories of BMI, including overweight and obese (OW = 25.0–29.9, OB ≥ 30) showed associations with milk FA composition when compared to the normal weight group (pBMI ≤ 24.9). The results indicated significant associations between BMI ≥ 30 and MUFA, n3-PUFA, n3-LCPUFA, and the n6/n3-LCPUFA ratio (Supplemental Table 5).

**FIGURE 3:**
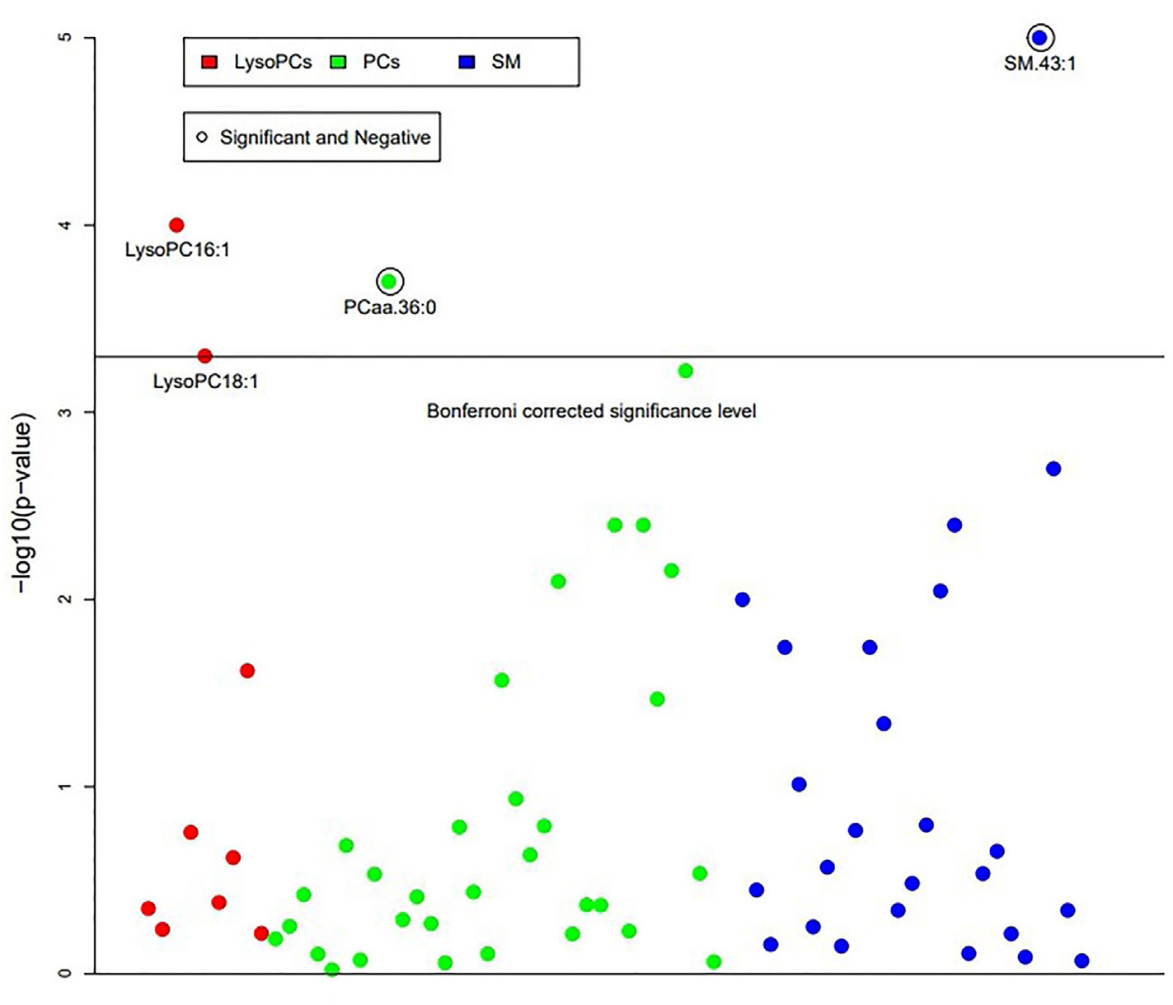
Adjusted associations of milk %phospholipid species with maternal prepregnancy Body mass index (pBMI kg/m²). *P* values of the multiple regression models relating maternal prepregnancy BMI to milk percentage PL (%PL). The dark horizontal line corresponds to the Bonferroni-corrected significance level of α =0.05/99 (number of analytes). Points are −log (*P* values) of the regression model. The independent variable is maternal prepregnancy BMI (pBMI kg/m²); the dependent variable is the respective %PL. Only significant %PL species are presented with their name, and significantly negatively associated %PL species are encircled.

In both unadjusted and adjusted models, pBMI showed no significant associations with the total of choline-containing PL classes (%LysoPC, %PC, %SM) (Supplemental Tables 6 and 7). However, pBMI was positively associated (*P* < 0.0005) with LysoPC16:1[β = 0.171, B(95% CI) = 0.001(0.001, 0.002), *P* < 0.0005], and LysoPC18:1, [β = 0.155, B(95% CI) = 0.005(0.002, 0.007), *P* < 0.0005] whereas pBMI showed significant negative associations with PCaa.36:0 [β = −0.152, B(95% CI) = −0.001(−0.002, −0.001), *P* < 0.0005] and SM.43:1 [β = −0.192, B(95%CI) = −0.003(−0.005, −0.002), *P* < 0.0005] after adjustment of study covariates (Figure 3, Supplemental Table 7). Adjusted associations with BMI categories (OW = 25.0–29.9, OB ≥ 30) and PL classes remained nonsignificant (Supplemental Table 8). Among covariates, maternal cod liver oil consumption and the number of fatty fish servings in days per year showed significant positive associations with n3-LCPUFA (C20:5n–3, C22:5n–3, C22:6n–3) and PC (PCaa36:5, PCaa38:5, PCaa38:6, PCaa40:6) (Supplemental Table 9).

The study results revealed significant variations in lipid metabolites using ANOVA testing and confirmed most of them through regression analysis. The regression analysis with and without outliers revealed no difference in significant associations (Supplemental Tables 3, 7, 10, and 11). However, influential outliers were characterized as model-fit outliers, indicating that their presence influenced the model’s fit [32]. Therefore, the results are reported without outliers, which provided higher R^2^-values. Although applying the CLR method to transform a subset of PL data resulted in lower significance levels for the associations observed with pBMI (Supplemental Tables 12 and 13), both statistical approaches yielded similar patterns of results, suggesting that the findings are not an artifact of statistical analysis.

## Discussion

Our findings from this observational study of Norwegian mothers’ milk indicate that maternal pBMI influences the levels of milk lipid metabolites and is associated with some human milk FA and choline-containing PL species percentages. Specifically, we found that maternal pBMI was significantly associated with the n6/n3-LCPUFA ratio, n3-LCPUFA, and MUFA with corresponding choline-containing PL species in human milk. Milk from mothers with obesity exhibited significantly lower levels of n3-LCPUFA while displaying a higher n6/n3-LCPUFA ratio, suggesting a proinflammatory profile [11].

Daniel et al. [40] reported a significant association between maternal BMI and the fat content of human milk in their meta-analysis, though the results had limited certainty due to the use of multiple quantification techniques [40]. In the current study, total fat content did not demonstrate significant differences or associations across BMI groups. Notably, the calculation of total fat content derived from the FA analysis is likely to underestimate the actual fat content due to excluding other lipid components such as glycerol and cholesterol. Additionally, the FA composition reported in this study is expressed as a percentage, which does not account for the absolute differences in total fat content.

Moreover, we found no significant differences in the percentage contribution of choline-containing PL classes (%LysoPC, %PC, and %SM) in BMI groups, suggesting that maternal pBMI does not substantially alter PL synthesis in the mammary gland.

Altered levels of n3 and n6-LCPUFA in mothers' milk with obesity have been found in other studies [12,35,41,42], which may have multiple explanations, including differences in dietary patterns, enzyme activity, and bioavailability of FA from adipose stores [[43], [44], [45], [46], [47]]. Dietary FA intake strongly predicts milk and plasma FA profiles with notable correlations between circulating and milk concentrations of many FAs, especially LCPUFA [43]. Furthermore, FA-desaturase activity (Δ-5 and Δ-6 desaturase) influences the levels of LCPUFA, which is also modulated by the dietary intake of preformed n3-LCPUFA [45,46,48]. It has also been demonstrated that most of the FAs in milk are transferred from internal stores, such as the liver and adipose tissues [3,47]. During early pregnancy, maternal body fat accumulation allows for significant amounts of FA to be stored, particularly LCPUFA, derived from both diet and maternal endogenous synthesis [48,49]. As gestation progresses, the accumulation of fat depots in maternal tissues decreases due to higher lipolysis and mobilization of TAG, leading to elevated levels of serum TAG, very low-density lipoprotein, and nonesterified FA, which in turn increases the bioavailability of FA for transfer to the fetus, including LCPUFA in the form of nonesterified FAs [49,50]. The rapid increase in lipoprotein lipase activity in mammary glands and its low activity in adipose tissue during the postpartum period facilitates increased uptake of circulating TAG by the mammary glands instead of their uptake by adipose tissue. These changes facilitate the uptake of diet-derived LCPUFA by the mammary gland and their availability to synthesize milk lipids [51,52]. However, previous research showed that maternal pBMI can modulate the bioavailability of LCPUFA, with higher pBMI associated with lower levels of n3 and elevated levels of n6-PUFA in maternal circulation during pregnancy and lactation [12]. Furthermore, other studies demonstrated that with increasing BMI and fat mass, the correlation of MUFA and n6-PUFA content between plasma nonesterified FAs and adipose tissue increases, possibly due to a higher contribution of these FA released from adipose tissue to the plasma nonesterified FA pool [53,54]. This suggests that in mothers with obesity, maternal circulation may primarily depend on the current dietary intake of LCPUFA to optimize human milk levels.

This study found no significant difference in the maternal intake of n3-LCPUFA (from cod liver oil consumption and fatty fish intake) among the pBMI groups. However, mothers with pBMI > 24.9 had significantly lower levels of n3-LCPUFA in their milk compared with mothers with normal weight. Recently, Walker et al. [55] have suggested that chronic inflammation in obesity suppresses lipoprotein lipase activity in the mammary gland that disrupts the transfer of long-chain FAs (LCFA > 16 carbon) from plasma to the mammary gland [55]. Since the mammary gland requires FA as an energy source and to synthesize TAG, the disrupted transfer of LCFA may adversely affect the nutritional quality of human milk. Similarities in diet among mothers in different BMI groups suggest that differences in milk composition among this study subgroup may be due to the above-mentioned metabolic differences rather than diet, which potentially influences the uptake of preformed n3-LCPUFA, leading to a significantly higher n6/n3-LCPUFA status. Further differences in enzyme function between lean and mothers with obesity may account for FA composition differences between the BMI groups. Increased stearoyl-CoA desaturase activity is associated with obesity [56], which can explain the positive association between pBMI and MUFA (C16:1–n7, C18:1–n7, C18:1–n9), as well as the corresponding LysoPC (Lysopc16:1, LysoPC18:1) and the concurrent negative association of PC36:0. Stearoyl-CoA desaturase plays a crucial role in endogenous MUFA synthesis and is considered a key regulator of lipid homeostasis, particularly in the liver and adipose tissue, where lipogenesis is most prevalent [57]. Hellmuth et al. [58] demonstrated strong positive associations between concentrations of LysoPC 16:1 and LysoPC 18:1 in cord blood with birth weight, suggesting a possible role of these LysoPC in early postnatal growth of offspring born to mothers with obesity.

Various studies have explored the link between obesity and SM in human subjects and murine models [59]. Serum-based research demonstrated that parameters of obesity and body composition are associated with a higher concentration of SM species with distinct saturated FA acyl chains (C18:0, C20:0, C22:0, and C24:0) [19,20]. Our study discovered overall nonsignificant negative associations between maternal pBMI and milk SM species, suggesting that the origin and metabolic regulation of SM in milk fat globular membranes differ from serum-based SM, which are components of lipoproteins. Among study covariates, maternal fish intake and cod liver oil consumption appear to be strongly associated with the composition of milk n3-LCPUFA, eicosapentaenoic acid (C20:5n-3), docosahexaenoic acid (22:6n-3), and corresponding PL species (PCaa36:5, PCaa38:5, PCaa38:6, PCaa40:6), indicating that maternal diet can affect milk FA and PL composition [7,60].

### Strengths and limitations

Our study is the first to explore the relationship between maternal pBMI and various individual FA and choline-containing PL species in human milk. The study included a large sample size, and most mothers were breastfeeding exclusively during the milk collection period. However, the low percentage (around 13%) of individuals with a pBMI above 30 kg/m² limited the power to identify obesity-related associations. We considered fatty fish and cod liver oil intake as potential sources of n3-LCPUFA composition. However, we recognized that our analysis would have benefited from a more comprehensive assessment of dietary exposures (ruminant products, fiber, MUFA intake). The study population was recruited from 5 counties across Norway, including northern, southern, western, and eastern regions. This study is the first to investigate the relationship between BMI and milk lipid composition in a population with high fish intake. However, the results may not be applicable to populations with different dietary habits or environmental conditions, although there seems to be a general agreement with studies in other areas. Our analytical method had limitations in identifying the exact molecular structure of metabolites, potentially restricting the scope of our findings. The linear regression models only explained small portions of the variation in human milk PL composition, indicating the presence of further predictors that could not be taken into account.

In conclusion, the study findings suggest that maternal pBMI is associated with milk FA and choline-containing PL species composition. Our finding of a negative association between pBMI and n3-LCPUFA is likely due to lower lipoprotein lipase activity rather than differences in dietary habits. MUFA and n6-LCPUFA could be higher with higher BMI because endogenous synthesis is increased. There are no indications for altered TAG and PL synthesis processes in subjects with obesity, and species composition of both lipid classes seems similarly affected by FA availability. Yet, the FA composition of diet does impact the composition of TAG and PL in mothers' milk across all BMI groups, allowing all mothers to improve their milk FA composition via diet or supplementation. In conclusion, improving maternal body weight and diet before and during pregnancy and lactation should be a priority, which may improve the nutritional quality of human milk and ultimately support infants' short and long-term development.

## Author contributions

The authors’ responsibilities were as follows – ME, HD and BK designed research; TAB conducted research; TBA and MT analyzed data; and TBA, RC and HD wrote the article. BK had primary responsibility for final content; and all authors: read and approved the final manuscript.

## Data availability

Data described in the manuscript, code book, and analytic code will be made available as far as in line with applicable data protection regulations and upon request pending application and approval.

## Funding

This work was financially supported in part by the Research Council of Norway, NEVRINOR [grant agreement no. 226402], and by the German Federal Ministry of Education and Research (BMBF) as part of the German Center for Child and Adolescent Health (DZKJ) (01GL2406A). BK is the Else Kröner Senior Professor of Paediatrics at LMU—the University of Munich, financially supported by the charitable Else Kröner-Fresenius-Foundation, LMU Medical Faculty, and LMU University Hospitals.

## Conflict of interest

The authors report no conflicts of interest.
