# Supplementary material for: The Associations of Maternal Prepregnancy Body Mass Index With Human Milk Fatty Acid and Phospholipid Composition in the Observational Norwegian Human Milk Study
Source: J Nutr. 2025 Apr 12;155(6):1818–27. doi: 10.1016/j.tjnut.2025.04.009 (PMC12264541; doi:10.1016/j.tjnut.2025.04.009)
Supplement: Multimedia component 1 [file mmc1.pdf]

Manuscript Title: The Associations of Maternal Pre-pregnancy Body Mass Index with Human Milk Fatty Acid and Phospholipid Composition in the Observational Norwegian Human Milk Study.

First Author: Talat Bashir Ahmed

Supplementary Figure 1:

Directed acyclic graph (DAG) model to explain the relationship between maternal pre-pregnancy obesity with human milk lipid (%FA, %PL) composition.

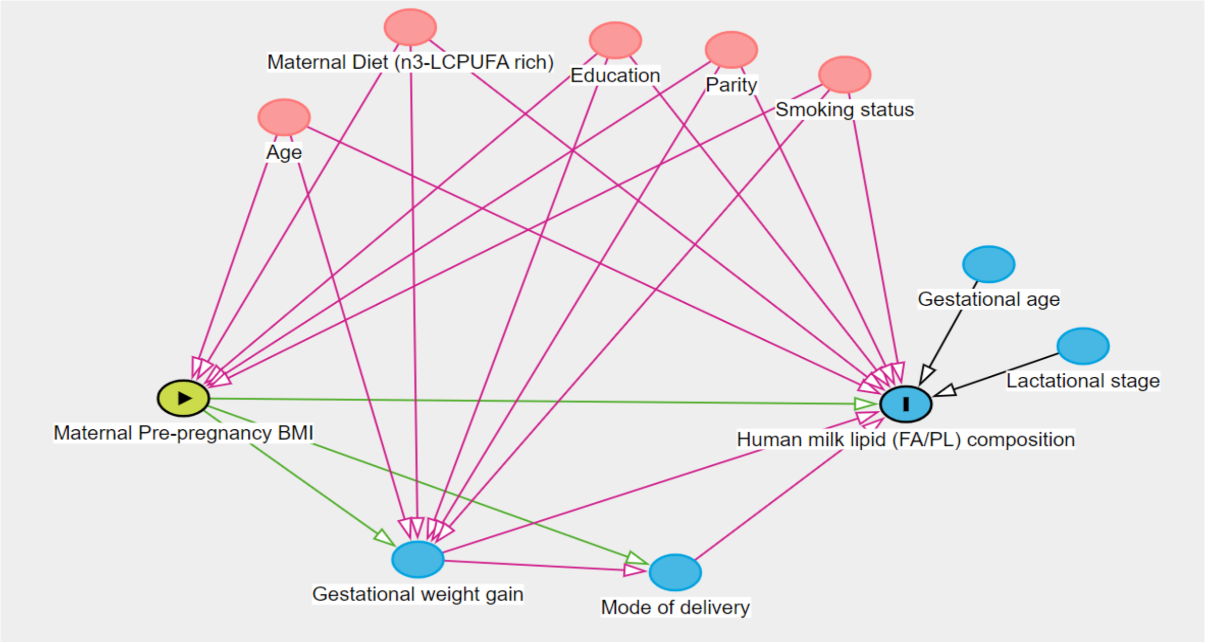

Each circle represents a variable, with lines indicating biasing paths (pink lines) and causal paths (green lines). Study Exposure, pre-pregnancy BMI (green circle), connected with the causal path to study Outcome, milk %FA, and %PL (blue circle). Study Covariates, maternal age, education, diet, parity, and smoking status (pink circles) connected with biasing paths to the study exposure and outcome. Study exposure (pBMI) connected with the causal path to study Mediators, excess gestational weight gain, and mode of delivery (blue circles), which further connected with the biasing path to study outcome (%FA, %PL). Gestational age and lactational stage (blue circles) indicate the ancestors of the study outcome.

Manuscript Title: The Associations of Maternal Pre-pregnancy Body Mass Index with Human Milk Fatty Acid and Phospholipid Composition in the Observational Norwegian Human Milk Study.

First Author: Talat Bashir Ahmed

Supplementary Table 1: Characteristics of Study Population (n=628)

**Maternal Characteristics**	n	*% or Mean(SD)
Maternal Age(Years)	628	29.49 (4.27)
Pre-pregnancy body mass index(kg/m²)	628	24.84 (4.57)
BMI ≤24.9 (Normal+underweight)	370	58.90
BMI=25.00 – 29.9 (Overweight)	177	28.20
BMI≥30 (Obese)	81	12.90
Gestational weight gain (kg)	628	15.22 (5.85)
Excess weight gain in pregnancy[1]	628	
Normal gestational weight gain	259	41.20
Excess gestational weight gain	369	58.80
Parity	628	
Primiparous	249	39.60
Multiparous	379	60.40
Mode of delivery	628	
Vaginal delivery	529	84.20
Cesarean section	99	15.80
Maternal Education	628	
<12 years	52	8.30
≥ 12 years	576	91.70
Maternal smoking status	628	
Never smoking	367	58.40
Past+current smoker	261	41.60
Maternal dietary habits (servings in days/year)	628	
Total meals of fatty fish dinner		25.83 (28.42)
Total lean fish as cod dinner		33.55 (31.32)
Vegetarian dinner		34.93 (39.50)
Cod liver oil		147.00 (163.18)
**Infant Characteristics**		
Gestational age (days)	628	280.19 (12.78)
Child's birth weight (gm)		3638.09 (592.57)
Child's sex		
Boy	327	52.10
Girl	301	47.90

Data expressed as mean ± SD for continuous variables and n (%) for categorical variables. [1] Defined per body mass index category according to Institutes of Medicine guidelines: for underweight women, >18.1 kg; for normal weight women, >15.9 kg; for overweight women, >11.3 kg; and for obese women, > 9.1 kg.

Manuscript Title: The Associations of Maternal Pre-pregnancy Body Mass Index with Human Milk Fatty Acid and Phospholipid Composition in the Observational Norwegian Human Milk Study.

First Author: Talat Bashir Ahmed

Supplementary Table 2: Distribution of %Phospholipid species profile in human milk samples (n=628) according to maternal pre-pregnancy BMI (pBMI kg/m²).

%PL	Total study samples (n=628) Mean (SD)	Normal-Weight (n=370) Mean (SD)	Overweight (n=177) Mean (SD)	Obesity (n=81) Mean (SD)	p-value
**%LysoPC**	4.388 (1.535)	4.378 (1.50)	4.415 (1.52)	4.373 (1.73)	0.962
lysoPC14:0	0.477 (0.220)	0.479 (0.221)	0.463 (0.210)	0.501 (0.233)	0.438
lysoPC16:0	2.309 (0.920)	2.313 (0.892)	2.337 (0.929)	2.235 (1.027)	0.710
lysoPC16:1	0.050 (0.029)	0.047 (0.027) [a]	0.054 (0.029) [b]	0.055 (0.038) [ab]	**0.009**
lysoPC18:0	0.356 (0.177)	0.358 (0.188)	0.354 (0.154)	0.349 (0.172)	0.907
lysoPC18:1	0.338 (0.135)	0.329 (0.132)	0.347 (0.136)	0.362 (0.147)	0.078
lysoPC18:2	0.730 (0.416)	0.727 (0.416)	0.731 (0.391)	0.745 (0.468)	0.942
lysoPC18:3	0.023 (0.015)	0.023 (0.015)	0.022 (0.014)	0.023 (0.016)	0.832
lysoPC20:4	0.051 (0.031)	0.049 (0.030)	0.053 (0.031)	0.053 (0.033)	0.274
lysoPC22:6	0.052 (0.031)	0.053 (0.031)	0.053 (0.033)	0.049 (0.034)	0.652
**%PC**	25.942 (5.652)	26.066 (5.68)	26.011 (5.55)	25.226 (5.75)	0.472
PCaa.30:0	0.940 (0.305)	0.958 (0.31)	0.893 (0.30)	0.959 (0.31)	0.055
PCaa.32:0	4.591 (1.08)	4.604 (1.08)	4.631 (1.10)	4.448 (1.06)	0.429
PCaa.32:1	0.402 (0.118)	0.401 (0.11)	0.412 (0.12)	0.389 (0.13)	0.341
PCaa.32:2	0.116 (0.042)	0.116 (0.04)	0.117 (0.04)	0.114 (0.04)	0.907
PCaa.32:3	0.017 (0.007)	0.017 (0.007)	0.017 (0.006)	0.016 (0.007)	0.254
PCaa.34:1	3.466 (0.996)	3.458 (1.00)	3.492 (1.00)	3.446 (0.961)	0.914
PCaa.34:2	2.211 (0.817)	2.213 (0.823)	2.231 (0.786)	2.157 (0.868)	0.797
PCaa.34:3	0.067 (0.027)	0.068 (0.028)	0.067 (0.028)	0.062 (0.024)	0.153
PCaa.36:0	0.163 (0.044)	0.167 (0.04) [a]	0.161 (0.05) **[ab]**	0.147 (0.04) [b]	**<0.001***
PCaa.36:1	1.970 (0.463)	1.957 (0.475)	2.004 (0.448)	1.952 (0.444)	0.505
PCaa.36:2	8.072 (2.435)	8.119 (2.477)	8.089 (2.346)	7.815 (2.447)	0.593
PCaa.36:3	0.760 (0.336)	0.776 (0.350)	0.743 (313)	0.723 (0.318)	0.329
PCaa.36:4	0.182 (0.088)	0.183 (0.089)	0.183 (0.085)	0.177 (0.090)	0.880
PCaa.36:5	0.020 (0.013)	0.021 (0.013)	0.020 (0.011)	0.018 (0.012)	0.102
PCaa.38:3	0.557 (0.157)	0.560 (0.157)	0.555 (0.147)	0.547 (0.176)	0.786
PCaa.38:4	0.439 (0.150)	0.439 (0.149)	0.444 (0.147)	0.430 (0.166)	0.769
PCaa.38:5	0.069 (0.040)	0.072 (0.04) [a]	0.067 (0.04) [ab]	0.057 (0.04) [b]	**0.008**
PCaa.38:6	0.039 (0.031)	0.042 (0.03) [a]	0.036 (0.03) [ab]	0.033 (0.03) [b]	**0.010**
PCaa.40:4	0.020 (0.009)	0.020 (0.009)	0.020 (0.009)	0.020 (0.009)	0.833
PCaa.40:5	0.041 (0.018)	0.041 (0.018)	0.040 (0.018)	0.038 (0.018)	0.284
PCaa.40:6	0.096 (0.05)	0.100 (0.05) [a]	0.095 (0.05) [ab]	0.078 (0.04) [b]	**0.003**
PCae.30:0	0.047 (0.023)	0.048 (0.023)	0.045 (0.020)	0.046 (0.029)	0.534
PCae.32:0	0.281 (0.081)	0.285 (0.083)	0.277 (0.078)	0.270 (0.078)	0.262
PCae.32:1	0.101 (0.030)	0.099 (0.028)	0.105 (0.032)	0.098 (0.030)	0.127
PCae.34:0	0.284 (0.083)	0.292 (0.09) [a]	0.281 (0.07) [ab]	0.257 (0.08) [b]	**0.002**
PCae.34:1	0.219 (0.061)	0.220 (0.062)	0.219 (0.062)	0.210 (0.057)	0.358
PCae.34:2	0.157 (0.042)	0.160 (0.044)	0.153 (0.037)	0.151 (0.044)	0.077
PCae.34:3	0.147 (0.041)	0.149 (0.040)	0.147 (0.042)	0.139 (0.046)	0.103

PCae.36:2	0.204 (0.058)	0.209 (0.06) [a]	0.201 (0.05) [ab]	0.184 (0.05) [b]	**0.002**
PCae.36:3	0.132 (0.041)	0.136 (0.04) [a]	0.129 (0.04) [ab]	0.122 (0.04) [b]	**0.006**
PCae.36:4	0.052 (0.025)	0.052 (0.025)	0.053 (0.026)	0.047 (0.027)	0.230
PCae.36:5	0.044 (0.018)	0.044 (0.018)	0.046 (0.018)	0.040 (0.019	0.057
PCae.38:3	0.039 (0.015)	0.040 (0.01) [a]	0.038 (0.01) [ab]	0.035 (0.01) [b]	**0.009**
**%SM**	69.670(6.14)	69.556 (6.16)	69.574 (5.99)	70.401 (6.39)	0.518
SM.33:1	0.177(0.051)	0.180 (0.052)	0.174 (0.050)	0.167 (0.052)	0.079
SM.34:1	5.679(0.962)	5.679 (0.941)	5.734 (0.978)	5.563 (1.021)	0.413
SM.34:2	0.175(0.074)	0.173 (0.071)	0.181 (0.074)	0.169 (0.088)	0.373
SM.35:1	0.341(0.085)	0.346 (0.087) [a]	0.341(0.081)[ab]	0.319 (0.086) [b]	**0.030**
SM.36:1	8.079(1.464)	7.986 (1.490)	8.275 (1.422)	8.075 (1.404)	0.096
SM.36:2	0.512(0.150)	0.507 (0.147)	0.532 (0.150)	0.490 (0.157)	0.064
SM.37:1	0.376(0.097)	0.378 (0.099) [ab]	0.382 (0.092) [a]	0.351 (0.097) [b]	**0.048**
SM.38:1	4.086(0.615)	4.098 (0.646)	4.048 (0.569)	4.117 (0.565)	0.602
SM.38:2	0.357(0.092)	0.355 (0.93)	0.362 (0.092)	0.352 (0.087)	0.595
SM.39:1	0.597(0.120)	0.603 (0.123)	0.595 (0.116)	0.576 (0.114)	0.183
SM.39:2	0.057(0.020)	0.057 (0.020)	0.056 (0.020)	0.053 (0.020)	0.133
SM.40:1	23.068(6.015)	22.906(5.856)	23.060 (6.001)	23.830(6.742)	0.457
SM.40:2	1.403(0.261)	1.412 (0.261)	1.405 (0.258)	1.361(0.264)	0.277
SM.40:3	0.077(0.026)	0.079 (0.027)	0.077 (0.026)	0.071(0.024)	0.056
SM.41:1	2.876(0.493)	2.906 (0.503)	2.831 (0.425)	2.836(0.578)	0.184
SM.41:2	0.576(0.137)	0.585 (0.138) [a]	0.574 (0.136)[a]	0.541(0.131) [b]	**0.036**
SM.42:1	12.007(2.219)	12.004 (2.189) [ab]	11.765(2.157)[b]	12.551(2.410) [a]	**0.030**
SM.42:2	7.560(1.672)	7.611 (1.665)	7.537 (1.761)	7.383(1.506)	0.526
SM.42:3	0.706(0.180)	0.713 (0.184)	0.702 (0.179)	0.682(0.164)	0.356
SM.42:4	0.040(0.011)	0.040 (0.012)	0.040 (0.010)	0.039(0.012)	0.500
SM.42:6	0.023(0.007)	0.023 (0.007)	0.022(0.006)	0.023(0.008)	0.515
SM.43:1	0.376(0.079)	0.384 (0.081) [a]	0.363 (0.071) [b]	0.368(0.084) [ab]	**0.011**
SM.43:2	0.347(0.094)	0.355 (0.095) [a]	0.343(0.094) [ab]	0.320(0.083) [b]	**0.008**
SM.44:2	0.146(0.043)	0.148 (0.044)	0.145 (0.044)	0.136(0.036)	0.079
SM.44:6	0.028(0.008)	0.029 (0.008) [ab]	0.027(0.008) [b]	0.030(0.008) [a]	**0.009**

%PL: percentage phospholipid composition, LysoPC: Lysophosphatidylcholine, PC: Phosphatidylcholine, PCaa: diacyl-phosphatidylcholine, PCae: acyl-alkyl phosphatidylcholine, SM: Sphingomyelin. Data presented as mean (SD). P-values are derived from one-way ANOVA and Bonferroni post-hoc test. Statistically significant p-values < 0.05 are highlighted in bold, and a star also marks those below the adjusted p-values. Adjusted p-value = 0.05/99 = 0.0005.

Manuscript Title: The Associations of Maternal Pre-pregnancy Body Mass Index with Human Milk Fatty Acid and Phospholipid Composition in the Observational Norwegian Human Milk Study.

First Author: Talat Bashir Ahmed

Supplementary Table 3: Adjusted[1] associations of %Fatty acids in human milk samples (n=628) with maternal pre-pregnancy body mass index (pBMI kg/m²).

%FA	R^2	B (95% CI)	β	Corr-Part	P-value
C8:0	0.040	-0.002 (-0.004 _ -0.0004)	-0.108	-0.096	**0.016**
C10:0	0.051	-0.005 (-0.011 _ 0.002)	-0.061	-0.054	0.169
C12:0	0.062	-0.016 (-0.046 _ 0.015)	-0.045	-0.040	0.308
C13:0	0.056	-0.002 (-0.002 _ -0.001)	-0.169	-0.151	**<0.001**
C14:0	0.060	-0.015 (-0.041 _ 0.011)	-0.049	-0.044	0.265
C15:0	0.099	-0.004 (-0.005 _ -0.002)	-0.186	-0.166	**<0.001**
C16:0	0.031	0.017 (-0.015 _ 0.049)	0.046	0.041	0.300
C17:0	0.052	-0.001 (-0.002 _ -0.0001)	-0.099	-0.088	**0.026**
C18:0	0.043	-0.033 (-0.056 _ -0.009)	-0.121	-0.108	**0.007**
C22:0	0.057	-0.001 (-0.002_ -0.0002)	-0.114	-0.101	**0.010**
C14:1	0.029	0.0004 (-0.001 _ 0.002)	0.034	0.030	0.451
C15:1	0.055	-0.0002 (-0.001 _ 0.00009)	-0.065	-0.058	0.143
C16:1t	0.037	0.00009 (-0.0003 _ 0.001)	0.018	0.016	0.682
C18:1t	0.040	-0.002 (-0.006 _ 0.001)	-0.057	-0.051	0.198
C22:1t	0.117	-0.001 (-0.001 _ -0.00009)	-0.098	-0.088	**0.021**
C16:1n-7	0.149	0.029 (0.018 _ 0.040)	0.212	0.189	**<0.001***
C18:1n-7	0.119	0.009 (0.005 _ 0.014)	0.195	0.174	**<0.001***
C18:1n-9	0.074	0.092 (0.046 _ 0.138)	0.171	0.152	**<0.001***
C20:1n-9	0.146	-0.003 (-0.004 _ -0.001)	-0.128	-0.114	**0.002**
C18:2n-6	0.036	-0.051 (-0.099 _ -0.002)	-0.092	-0.082	**0.040**
C18:3n-6	0.043	0.001 (-0.0002 _ 0.001)	0.060	0.054	0.176
C18:3n-3	0.042	-0.004 (-0.010 _ 0.001)	-0.070	-0.062	0.115
C20:2n-6	0.042	-0.002 (-0.005 _ 0.0002)	-0.080	-0.071	0.073
C20:3n-6	0.058	0.001 (-0.0004 _ 0.002)	0.059	0.052	0.182
C20:3n-9	0.052	-0.00003 (-0.0001 _ 0.00009)	-0.023	-0.020	0.606
C20:3n-3	0.063	-0.0002 (-0.0004 _ -0.00002)	-0.098	-0.087	**0.026**
C20:4n-6	0.055	0.001 (-0.001_ 0.002)	0.050	0.045	0.254
C20:5n-3	0.329	-0.002 (-0.003_ -0.001)	-0.132	-0.118	**<0.001***
C22:4n-6	0.103	0.001 (0.0002 _ 0.001)	0.150	0.134	**<0.001***
C22:5n-6	0.027	-0.0001 (-0.0004_ 0.0001)	-0.052	-0.046	0.246
C22:5n-3	0.284	-0.001 (-0.002 _ -0.001)	-0.118	-0.105	**0.002**
C22:6n-3	0.340	-0.006 (-0.010 _ -0.003	-0.136	-0.121	**<0.001***
SFA	0.058	-0.061 (-0.128 _ 0.007)	-0.078	-0.069	0.077
MCSFA	0.062	-0.037 (-0.098 _ 0.023)	-0.053	-0.047	0.225
LCSFA	0.035	-0.017 (-0.059 _ 0.026)	-0.035	-0.031	0.437
MUFA	0.101	0.128 (0.076 _ 0.180)	0.207	0.185	**<0.001***
ODFA	0.097	-0.006 (-0.009 _ -0.004)	-0.200	-0.178	**<0.001**
tFA	0.036	-0.003 (-0.007 _0.001)	-0.067	-0.060	0.132
PUFA	0.037	-0.065 (-0.119 _ -0.010)	-0.104	-0.092	**0.020**
N6-PUFA	0.035	-0.050 (-0.101 _ -0.0001)	-0.088	-0.078	**0.049**
N3-PUFA	0.217	-0.014 (-0.022 _ -0.006)	-0.138	-0.123	**<0.001**

LCPUFA	0.215	-0.008 (-0.014 _ -0.001)	-0.098	-0.087	**0.015**
N6-LCPUFA	0.059	0.002 (-0.0003 _ 0.005)	0.075	0.066	0.090
N3-LCPUFA	0.340	-0.010 (-0.015 _ -0.005)	-0.138	-0.123	**<0.001***
N6/N3 PUFA	0.239	0.031 (-0.001 _ 0.063)	0.075	0.067	0.059
N6/N3-LCPUFA	0.395	0.020 (0.012 _ 0.028)	0.170	0.152	**<0.001***
Total FA (g/l)	0.059	0.285 (0.120 _ 0.449)	0.150	0.133	**<0.001**

[1]Model adjusted for maternal age, gestational weight gain, parity, maternal fish and cod liver oil consumption, smoking, education, and mode of delivery. Maternal pre-pregnancy BMI kg/m²: study exposure (independent variable), %FA: outcome variable (dependent variable). R^2: coefficient of determination; B(%CI): unstandardized coefficient (95% confidence interval); β: standardized regression coefficient. Statistically significant p-values < 0.05 are highlighted in bold. Associations below the adjusted p-values are additionally marked by a star. Adjusted p-value = 0.05/99 = 0.0005**.**

Manuscript Title: The Associations of Maternal Pre-pregnancy Body Mass Index with Human Milk Fatty Acid and Phospholipid Composition in the Observational Norwegian Human Milk Study.

First Author: Talat Bashir Ahmed

Supplementary Table 4: Unadjusted associations of %Fatty acids in human milk samples (n = 628) with maternal pre-pregnancy body mass index (pBMI, kg/m²).

%FA	B (95% CI)	β	P-value
C8:0	-0.001 (-0.002 _ 0.001)	-0.037	0.358
C10:0	-0.001 (-0.007 _ 0.005)	-0.014	0.727
C12:0	0.004 (-0.024 _ 0.031)	0.011	0.787
C13:0	-0.001 (0.003 _ -0.002)	-0.117	**0.003**
C14:0	0.0002 (-0.024 _ 0.024)	0.001	0.985
C15:0	-0.003 (-0.005 _ -0.002)	-0.156	**<0.001***
C16:0	0.021 (-0.008 _ 0.049)	0.057	0.155
C17:0	-0.001 (-0.002 _ -0.0003)	-0.104	**0.009**
C18:0	-0.022 (-0.043 _ -0.001)	-0.082	**0.039**
C22:0	-0.001 (-0.001 _ 0.0001)	-0.064	0.108
C14:1	-0.0001 (-0.001 _ 0.001)	-0.012	0.770
C15:1	-0.0003 (-0.001 _ -0.00005)	-0.079	**0.047**
C16:1t	-0.00003 (-0.0004 _0.0003)	-0.008	0.850
C18:1t	-0.003 (-0.006 _ 0.0002)	-0.073	0.067
C22:1t	-0.001 (-0.001 _ -0.0002)	-0.112	**0.005**
C16:1n-7	0.015 (0.005 _ 0.026)	0.112	**0.005**
C18:1n-7	0.005 (0.001_ 0.009)	0.106	**0.008**
C18:1n-9	0.060 (0.018 _ 0.102)	0.112	**0.005**
C20:1n-9	-0.003 (-0.005 _ -0.002)	-0.152	<**0.001***
C18:2n-6	-0.049 (-0.092 _ -0.006)	-0.088	**0.027**
C18:3n-6	0.0002 (-0.0003_ 0.001)	0.033	0.409
C18:3n-3	-0.005 (-0.01 _ -0.00004)	-0.078	**0.050**
C20:3n-3	-0.0002 (-0.0004 _ -0.000008)	-0.081	**0.041**
C20:2n-6	-0.002 (-0.004 _ 0.001)	-0.061	0.126
C20:3n-6	0.001 (-0.00002 _ 0.003)	0.077	0.054
C20:3n-9	-0.00001 (-0.0001 _ 0.00009)	-0.009	0.816
C20:4n-6	0.0001 (-0.001 _ 0.001)	0.008	0.848
C20:5n-3	-0.003 (-0.004 _ -0.002)	-0.193	**<0.001***
C22:4n-6	0.01 (0.0003 _ 0.001)	0.148	**<0.001***
C22:5n-6	-0.0001 (-0.0004 _ 0.00005)	-0.061	0.129
C22:5n-3	-0.002 (-0.003 _ -0.001)	-0.178	**<0.001***
C22:6n-3	-0.010 (-0.013 _ -0.006)	-0.211	**<0.001***
SFA	-0.005 (-0.066 _ 0.056)	-0.007	0.870
MUFA	0.077 (0.029 _ 0.125)	0.125	**0.002**

ODFA	-0.005 (-0.008 _ -0.003)	-0.167	**<0.001***
PUFA	-0.068 (-0.117 _ -0.020)	-0.110	**0.006**
N3-PUFA	-0.020 (-0.028 _ -0.012)	-0.192	**<0.001***
N6-PUFA	-0.048 (-0.093 _ -0.004)	-0.084	**0.034**
LCPUFA	-0.013 (-0.019 _ -0.007)	-0.168	**<0.001***
N3-LCPUFA	-0.002 (-0.004 _ -0.0003)	-0.093	**0.019**
N6-LCPUFA	0.002 (0.001 _ 0.003)	0.108	**0.007**
N6/N3-PUFA	0.052 (0.020 _ 0.084)	0.125	**0.002**
N6/N3-LCPUFA	0.027 (0.018 _ 0.036)	0.231	**<0.001***
Total FA (g/l)	0.184 (0.036 _ 0.333)	0.097	**0.015**

Maternal pre-pregnancy BMI kg/m²: study exposure (independent variable), %FA: outcome variable (dependent variable). B(%CI): unstandardized coefficient (95% confidence interval); β: standardized regression coefficient. Statistically significant p-values < 0.05 are highlighted in bold. Associations below the adjusted p-values are additionally marked by a star. Adjusted p-value = 0.05/99 = 0.0005.

Manuscript Title: The Associations of Maternal Pre-pregnancy Body Mass Index with Human Milk Fatty Acid and Phospholipid Composition in the Observational Norwegian Human Milk Study.

First Author: Talat Bashir Ahmed

Supplementary Table 5: Adjusted[1] associations of %Fatty acids in human milk samples (n = 628) with maternal pre-pregnancy body mass index (pBMI kg/m²) categories (†OW-Overweight = 25.0-29.9, ‡OB-Obese ≥ 30) with reference to normal weight group (BMI ≤ 24.9kg/m²).

%FA	R^2	B(95%CI)	β	Corr-Part	P-value
C8:0	0.039	-0.017(-0.034_-0.0002)	-0.084	-0.079	0.047
		-0.023(-0.047_-0.002)	-0.082	-0.072	0.071
C10:0	0.060	-0.088(-0.152_-0.024)	-0.113	-0.106	0.007
		-0.066(-0.158_0.027)	-0.063	-0.055	0.164
C12:0	0.070	-0.375(-0.668_-0.083)	-0.105	-0.098	0.012
		-0.229(-0.651_0.192)	-0.048	-0.042	0.286
C13:0	0.047	-0.008(-0.016_0.0004)	-0.078	-0.074	0.063
		-0.016(-0.028_-0.004)	-0.121	-0.106	0.007
C14:0	0.068	-0.326(-0.577_-0.075)	-0.106	-0.100	0.011
		-0.125(-0.487_0.238)	-0.030	-0.026	0.500
C15:0	0.094	-0.015(-0.031_0.001)†	-0.077†	-0.072	†0.062
		-0.045(-0.068_-0.022)‡	-0.167‡	-0.146	‡**<0.001***
C16:0	0.040	0.334(0.026_0.642)	0.090	0.084	0.034
		0.421(-0.024_0.865)	0.084	0.074	0.064
C17:0	0.056	0.001(-0.007_0.009)	0.012	0.012	0.766
		-0.015(-0.026_-0.003)	-0.112	-0.097	0.013
C18:0	0.043	-0.256(-0.483_-0.028)	-0.093	-0.087	0.028
		-0.343(-0.671_-0.015)	-0.093	-0.081	0.041
C22:0	0.052	-0.007(-0.015_0.001)	-0.074	-0.069	0.079
		-0.008(-0.020_0.004)	-0.059	-0.051	0.191
C14:1	0.031	0.007(-0.005_0.019)	0.047	0.044	0.272
		-0.002(-0.020_0.015)	-0.013	-0.011	0.781
C15:1	0.054	-0.002(-0.004_0.003)	-0.006	-0.005	0.890
		-0.004(-0.009_0.002)	-0.061	-0.053	0.179
C16:1n-7	0.145	0.231(0.123_0.339)†	0.167†	0.157	†**<0.001***
		0.269(0.114_0.425)‡	0.145‡	0.127	‡**<0.001**
C18:1n-7	0.119	0.080(0.041_0.119)†	0.162†	0.152	†**<0.001***
		0.093(0.036_0.149)‡	0.140‡	0.122	‡**0.001**
C18:1n-9	0.069	0.505(0.058_0.953)	0.092	0.087	0.027
		1.04(0.401_1.69)	0.142	0.124	**0.002**
C16:1t	0.039	-0.002(-0.006_0.003)	-0.031	-0.029	0.460
		-0.003(-0.009_0.003)	-0.042	-0.037	0.355
C18:1t	0.046	-0.006(-0.040_0.027)	-0.015	-0.014	0.714
		-0.057(-0.106_-0.009)	-0.106	-0.092	0.020
C22:1t	0.122	-0.001(-0.007_0.004)	-0.022	-0.021	0.585
		-0.012(-0.019_-0.004)	-0.131	-0.115	0.003

C18:2n-6	0.035	-0.047(-0.515_0.420)	-0.008	-0.008	0.842
		-0.602(-1.27_0.072)	-0.080	-0.070	0.080
C18:3n-6	0.048	0.007(-0.0001_0.014)	0.081	0.076	0.056
		-0.003(-0.014_0.007)	-0.027	-0.023	0.552
C20:2n-6	0.043	-0.020(-0.046_0.007)	-0.062	-0.058	0.141
		-0.033(-0.071_0.005)	-0.078	-0.068	0.088
C20:3n-6	0.058	0.009(-0.005_0.024)	0.054	0.051	0.194
		-0.00004(-0.021_0.020)	-0.0001	-0.0001	0.997
C20:4n-6	0.060	0.013(0.0003_0.025)	0.084	0.079	0.044
		0.0003(-0.017_0.018)	0.002	0.001	0.971
C22:4n-6	0.106	0.007(0.003_0.011)†	0.150†	0.141	**†<0.001**
		0.005(-0.002_0.011)‡	0.082‡	0.072	**‡0.061**
C22:5n-6	0.026	0.001(-0.002_0.003)	0.032	0.030	0.449
		-0.0003(-0.004_0.003)	-0.008	-0.007	0.857
C18:3n-3	0.046	0.0004(-0.052_0.053)	0.001	0.001	0.986
		-0.082(-0.158_-0.006)	-0.096	-0.084	0.034
C20:3n-3	0.065	-0.0003(-0.002_0.002)	-0.013	-0.013	0.747
		-0.004(-0.007_-0.001)	-0.109	-0.095	0.015
C20:5n-3	0.328	-0.004(-0.014_0.003)†	-0.023†	-0.022	†0.507
		-0.026(-0.042_-0.011)‡	-0.129‡	-0.113	**‡<0.001***
C22:5n-3	0.289	-0.00005(-0.009_0.008)†	-0.0005†	-0.0005	†0.989
		-0.022(-0.035_-0.010)‡	-0.140‡	-0.122	**‡<0.001***
C22:6n-3	0.339	-0.015(-0.047_0.017)†	-0.032†	-0.030	†0.367
		-0.083(-0.130_-0.037‡	-0.134‡	-0.117	**‡<0.001***
SFA	0.061	-0.758(-1.40_-0.110)	-0.096	-0.090	0.022
		-0.448(-1.38_0.487)	-0.042	-0.037	0.347
MCSFA	0.071	-0.807(-1.39_-0.22)	-0.113	-0.106	0.007
		-0.442(-1.28_0.399)	-0.046	-0.040	0.302
ODCFA	0.090	-0.022(-0.047_0.004)†	-0.069†	-0.065	†0.093
		-0.075(-0.112_-0.039)‡	-0.178‡	-0.155	**‡<0.001**
MUFA	0.094	0.816(0.310_1.32)†	0.130†	0.122	**†0.002**
		1.372(0.642_2.10)‡	0.163‡	0.142	**‡<0.001***
tFA	0.044	-0.009(-0.045_0.026)	-0.022	-0.020	0.606
		-0.072(-0.123_-0.021)	-0.125	-0.109	0.006
PUFA	0.037	-0.048(-0.574_0.478)	-0.008	-0.007	0.858
		-0.852(-1.61_-0.093)	-0.100	-0.087	0.028
N6-PUFA	0.034	-0.030(-0.515_0.456)	-0.005	-0.005	0.904
		-0.633(-1.33_0.067)	-0.081	-0.072	0.076
N3-PUFA	0.221	-0.018(-0.097_0.060)†	-0.017†	-0.016	†0.648
		-0.218(-0.331_-0.105)‡	-0.155‡	-0.135	**‡<0.001***
LCPUFA	0.222	0.012(-0.047_0.070)	0.015	0.014	0.698
		-0.132(-0.216_-0.047)	-0.125	-0.109	0.002
N6-LCPUFA	0.064	0.030(0.005_0.056)	0.097	0.091	0.021
		0.005(-0.032_0.042)	0.012	0.011	0.783
N3-LCPUFA	0.340	-0.019(-0.068_0.031)†	-0.026†	-0.024	†0.462
		-0.136(-0.208_-0.064)‡	-0.140‡	-0.122	**‡<0.001***
N6/N3-LCPUFA	0.391	0.080(0.0004_0.159)†	0.066†	0.062	†0.049
		0.255(0.141_0.370)‡	0.158‡	0.138	**‡<0.001***
Total FA	0.048	1.37(-0.224_2.96)	0.071	0.067	0.092
		2.01(-0.28_4.31)	0.078	0.068	0.086

BMI-categories (OW† Overweight = 25.0-29.9, OB‡ obese ≥ 30 kg/m²). [1]Model adjusted for maternal age, gestational weight gain, parity, maternal fish and cod liver oil consumption, smoking, education, and mode of delivery. Maternal pre-pregnancy BMI kg/m²: study exposure (independent variable), %FA: outcome variable

(dependent variable). R^2: coefficient of determination; B(%CI): unstandardized coefficient (95% confidence interval); β: standardized regression coefficient. Statistically significant p-values < 0.05 are highlighted in bold. Associations below the adjusted p-values are additionally marked by a star. Adjusted p-value = 0.05/99 = 0.0005**.**

Manuscript Title: The Associations of Maternal Pre-pregnancy Body Mass Index with Human Milk Fatty Acid and Phospholipid Composition in the Observational Norwegian Human Milk Study.

First Author: Talat Bashir Ahmed

Supplementary Table 6: Unadjusted associations of %Phospholipid species in human milk samples (n=628) with maternal pre-pregnancy body mass index (pBMI kg/m²).

%PL	B (95% CI)	β	P-value
LysoPC14:0	0.002 (-0.002 _ 0.006)	0.047	0.240
LysoPC16:0	-0.003 (-0.019 _ 0.013)	-0.016	0.687
LysoPC18:0	-0.002 (-0.006 _ 0.001)	-0.061	0.175
LysoPC16:1	0.001 (0.0003 _ 0.001)	0.135	**<0.001**
LysoPC18:1	0.004 (0.002 _ 0.006)	0.132	**<0.001**
LysoPC18:2	0.001 (-0.006 _ 0.008)	0.011	0.784
LysoPC18:3	0.00007 (-0.0001 _ 0.0003)	0.024	0.551
LysoPC20:4	0.0003 (-0.0001 _ 0.001)	0.052	0.193
LysoPC22:6	-0.0001 (-0.001 _ 0.0004)	-0.021	0.600
LysoPC	0.002 (-0.024 _ 0.029)	0.007	0.854
PCaa.30:0	-0.0003 (-0.006 _ 0.005)	-0.005	0.893
PCaa.32:0	-0.005 (-0.023 _ 0-014)	-0.020	0.626
PCaa.32:1	0.0002 (-0.002_ 0.002)	0.010	0.797
PCaa.32:2	-0.0001 (-0.001_ 0.001)	-0.016	0.688
PCaa.32:3	-0.00003 (-0.0001 _ 0.00008)	-0.023	0.566
PCaa.34:1	0.004 (-0.014 _ 0.021)	0.016	0.687
PCaa.34:2	-0.003 (-0.017 _ 0.011)	-0.014	0.718
PCaa.34:3	-0.0003 (-0.001 _ 0.0001)	-0.061	0.126
PCaa.36:0	-0.001 (-0.002 _ -0.001)	-0.141	**<0.001***
PCaa.36:1	0.001 (-0.007 _ 0.009)	0.010	0.809
PCaa.36:2	-0.027 (-0.069 _ 0.014)	-0.051	0.198
PCaa.36:3	-0.003 (-0.009 _ 0.003)	-0.044	0.270
PCaa.36:4	-0.0002 (-0.002 _ 0.001)	-0.013	0.751
PCaa.36:5	-0.0001 (-0.0004 _ 0.00001)	-0.073	0.068
PCaa.38:3	-0.001 (-0.004 _ 0.001)	-0.038	0.341
PCaa.38:4	-0.0004 (-0.003 _ 0.002)	-0.013	0.748
PCaa.38:5	-0.001(-0.002 _ -0.0004)	-0.122	**0.002**
PCaa.38:6	-0.001 (-0.001 _ -0.0003)	-0.106	**0.008**
PCaa.40:4	-0.00002 (-0.0001 _ 0.0001)	-0.013	0.736
PCaa.40:5	-0.0002 (-0.001 _ 0.00001)	-0.074	0.065
PCaa.40:6	-0.001 (-0.002 _ -0.001)	-0.133	**<0.001**
PCae.30:0	-0.00003 (-0.0004 _ 0.0003)	-0.008	0.850
PCae.32:0	-0.001 (-0.002 _ 0.0004)	-0.054	0.180
PCae.32:1	0.0001 (-0.0003 _ 0.001)	0.025	0.531

PCae.34:0	-0.002 (-0.004 _ -0.001)	-0.138	**<0.001**
PCae.34:1	-0.0003 (-0.001 _ 0.001)	-0.028	0.487
PCae.34:2	-0.001 (-0.001_-0.00003)	-0.082	**0.040**
PCae.34:3	-0.001 (-0.001 _ 0.00006)	-0.071	0.075
PCae.36:2	-0.002 (-0.003 _ -0.001)	-0.124	**0.002**
PCae.36:3	-0.001 (-0.002 _ -0.0002)	-0.110	**0.006**
PCae.36:4	-0.0002 (-0.001 _ 0.0002)	-0.037	0.360
PCae.36:5	-0.00002 (-0.0003 _ 0.0002)	-0.007	0.852
PCae.38:3	-0.0003 (-0.001 _ -0.0001)	-0.120	**0.003**
PC	-0.049 (-0.146 _ 0.048)	-0.040	0.317
SM.33:1	-0.001 (-0.002 _ -0.0003)	-0.107	**0.007**
SM.34:1	-0.010 (-0.027 _ 0.006)	-0.048	0.229
SM.34:2	0.00002 (-0.001 _ 0.001)	0.001	0.975
SM.35:1	-0.002 (-0.004 _ -0.001)	-0.118	**0.003**
SM.36:1	0.013 (-0.012 _ 0.038)	0.040	0.317
SM.36:2	-0.0002 (-0.003 _ 0.002)	-0.006	0.877
SM.37:1	-0.002 (-0.003 _-0.0002)	-0.080	**0.045**
SM.38:1	-0.001 (-0.012 _ 0.009)	-0.010	0.796
SM.38:2	0.0003 (-0.001 _ 0.002)	0.018	0.649
SM.39:1	-0.003 (-0.005 _ -0.001)	-0.105	**0.009**
SM.39:2	-0.0003 (-0.001 _ -0.00004)	-0.089	**0.026**
SM.40:1	0.074 (-0.029 _ 0.177)	0.057	0.157
SM.40:2	-0.004 (-0.008 _ 0.001)	-0.066	0.100
SM.40:3	-0.0003 (-0.001 _ 0.00005)	-0.068	0.089
SM.41:1	-0.009 (-0.017_-0.001)	-0.080	**0.045**
SM.41:2	-0.004 (-0.006 _ -0.002)	-0.134	**<0.001**
SM.42:1	0.024 (-0.014 _ 0.062)	0.050	0.210
SM.42:2	-0.020 (-0.049 _ 0.008)	-0.056	0.162
SM.42:3	-0.002 (-0.005 _ 0.001)	-0.058	0.149
SM.42:4	-0.00007 (-0.0002 _ 0.0001)	-0.032	0.420
SM.42:6	-0.000008 (-0.0001 _ 0.0001)	0.006	0.885
SM.43:1	-0.002 (-0.003 _ -0.001)	-0.120	**0.003**
SM.43:2	-0.003 (-0.004 _ -0.001)	-0.136	**<0.001**
SM.44:2	-0.001 (-0.001 _ 0.00003)	-0.074	0.063
SM.44:6	0.00002 (-0.0001 _ 0.0001)	0.013	0.740
SM	0.047 (-0.058 _ 0.152)	0.035	0.382

Maternal pre-pregnancy BMI kg/m²: study exposure (independent variable), %PL: percentage phospholipid composition as outcome variable (dependent variable), LysoPC: Lysophosphatidylcholine, PC: Phosphatidylcholine, PCaa: diacyl phosphatidylcholine, PCae: acyl-alkyl phosphatidylcholine, SM: sphingomyelin. B(%CI): unstandardized coefficient (95% confidence interval); β: standardized regression coefficient. Statistically significant p-values < 0.05 are highlighted in bold. Associations below the adjusted p-values are additionally marked by a star. Adjusted p-value = 0.05/99 = 0.0005**.**

Manuscript Title: The Associations of Maternal Pre-pregnancy Body Mass Index with Human Milk Fatty Acid and Phospholipid Composition in the Observational Norwegian Human Milk Study.

First Author: Talat Bashir Ahmed

Supplementary Table 7: Adjusted[1] associations of %Phospholipid species in human milk samples (n=628) with maternal pre-pregnancy body mass index (pBMI kg/m²).

**%PL**	**R^2**	**B (95% CI)**	**β**	**Corr-Part**	**P-value**
LysoPC14:0	0.052	0.002 (-0.003 _ 0.006)	0.034	0.030	0.447
LysoPC16:0	0.027	0.005 (-0.013 _ 0.023)	0.025	0.022	0.578
LysoPC16:1	0.053	0.001 (0.001 _ 0.002)	0.171	0.152	**<0.001***
LysoPC18:0	0.021	-0.002 (-0.006 _ 0.001)	-0.061	-0.054	0.175
LysoPC18:1	0.048	0.005 (0.002_ 0.007)	0.155	0.138	**<0.001***
LysoPC18:2	0.030	0.003 (-0.005 _ 0.011)	0.036	0.032	0.415
LysoPC18:3	0.031	0.0001 (-0.0001 _ 0.0004)	0.053	0.047	0.239
LysoPC20:4	0.039	0.001 (0.0001 _ 0.001)	0.101	0.090	**0.024**
LysoPC22:6	0.043	0.0001 (-0.0004 _ 0.001)	0.023	0.021	0.607
LysoPC	0.026	0.014 (-0.015 _ 0.044)	0.042	0.038	0.343
PCaa.30:0	0.022	-0.001 (-0.007_ 0.005)	-0.020	-0.018	0.649
PCaa.32:0	0.027	0.006 (-0.015 _ 0.027)	0.026	0.023	0.556
PCaa.32:1	0.038	0.001 (-0.001 _ 0.003)	0.039	0.035	0.377
PCaa.32:2	0.028	-0.0001 (-0.001 _ 0.001)	-0.012	-0.011	0.783
PCaa.32:3	0.041	-0.00004 (-0.0001 _ 0.0001)	0.003	0.002	0.950
PCaa.34:1	0.052	0.012 (-0.007 _ 0.031)	0.056	0.050	0.206
PCaa.34:2	0.045	0.002 (-0.014 _ 0.017)	0.009	0.008	0.842
PCaa.34:3	0.035	-0.0002 (-0.001 _ 0.0002)	-0.047	-0.042	0.293
PCaa.36:0	0.059	-0.001 (-0.002 _ -0.001)	-0.152	-0.144	**<0.001***
PCaa.36:1	0.034	0.003 (-0.006 _ -0.012)	0.029	0.026	0.513
PCaa.36:2	0.034	-0.021 (-0.067 _ 0.026)	-0.039	-0.034	0.387
PCaa.36:3	0.036	-0.002 (-0.008 _ 0.004)	-0.027	-0.024	0.538
PCaa.36:4	0.047	0.0001 (-0.002 _ 0.002)	0.007	0.006	0.872
PCaa.36:5	0.091	-0.0001 (-0.0003 _ 0.0005)	-0.060	-0.054	0.164
PCaa.38:3	0.052	-0.001 (-0.004 _ 0.002)	-0.040	-0.036	0.365
PCaa.38:4	0.063	0.0004 (-0.002 _ 0.003)	0.012	0.011	0.780
PCaa.38:5	0.129	-0.001 (-0.002 _ -0.0001)	-0.094	-0.084	**0.027**
PCaa.38:6	0.123	-0.0004 (-0.001 _ 0.0001)	-0.067	-0.060	0.116
PCaa.40:4	0.054	-0.0001 (-0.0002_0.00007)	-0.053	-0.047	0.231
PCaa.40:5	0.039	-0.0002 (-0.001 _ 0.0001)	-0.062	-0.055	0.162
PCaa40:6	0.140	-0.001 (-0.002 _ -0.0003)	-0.083	-0.074	**0.048**
PCae.30:0	0.024	-0.0001 (-0.001 _ 0.0003)	-0.023	-0.020	0.611
PCae.32:0	0.022	-0.001 (-0.002 _ 0.001)	-0.036	-0.032	0.427
PCae.32:1	0.052	0.0002 (-0.0003 _ 0.001)	0.035	0.031	0.429
PCae.34:0	0.076	-0.002 (-0.004 _ -0.001)	-0.128	-0.114	**0.004**

PCae.34:1	0.034	-0.0003 (-0.001 _ 0.001)	-0.024	-0.021	0.590
PCae.34:2	0.062	-0.001 (-0.002 _ -0.0003)	-0.125	-0.112	**0.004**
PCae.34:3	0.060	-0.001 (-0.002 _ -0.00005)	-0.094	-0.083	**0.034**
PCae.36:2	0.045	-0.002 (-0.003 _ 0.0004)	-0.120	-0.107	**0.007**
PCae.36:3	0.070	-0.001 (-0.002 _ -0.001)	-0.151	-0.135	**<0.001**
PCae.36:4	0.038	-0.0002 (-0.001 _ 0.0002)	-0.047	-0.042	0.290
PCae.36:5	0.041	0.00003 (-0.0003 _ 0.0003)	0.008	0.007	0.860
PCae.38:3	0.031	-0.0004 (-0.001 _ -0.0001)	-0.139	-0.124	**0.002**
PC	0.047	-0.014 (-0.121 _ 0.093)	-0.011	-0.010	0.798
SM.33:1	0.046	-0.001 (-0.002 _ -0.0003)	-0.114	-0.102	**0.010**
SM.34:1	0.032	-0.009 (-0.027 _ 0.010)	-0.041	-0.037	0.356
SM.34:2	0.035	0.0002 (-0.001 _ 0.002)	0.017	0.015	0.696
SM.35:1	0.048	-0.002 (-0.004 _-0.0003)	-0.105	-0.094	**0.018**
SM.36:1	0.086	0.023 (-0.004 _ 0.050)	0.072	0.064	0.097
SM.36:2	0.051	001 (-0.002 _ 0.004)	0.026	0.023	0.560
SM.37:1	0.046	-0.001 (-0.003 _ 0.001)	-0.049	-0.045	0.269
SM.38:1	0.027	-0.002 (-0.014 _ 0.010)	-0.017	-0.015	0.710
SM.38:2	0.026	0.001 (-0.001 _ 0.003)	0.061	0.055	0.171
SM.39:1	0.072	-0.003 (-0.005 _-0.0004)	-0.103	-0.092	**0.018**
SM.39:2	0.023	-0.0003 (-0.001 _ -0.000006)	-0.089	-0.080	**0.046**
SM.40:1	0.044	0.043 (-0.071_0.158)	0.033	0.029	0.457
SM.40:2	0.037	-0.002 (-0.007 _ 0.002)	-0.044	-0.039	0.328
SM.40:3	0.033	-0.0003 (-0.001 _ 0.0001)	-0.063	-0.056	0.160
SM.41:1	0.043	-0.013 (-0.022 _ -0.003)	-0.116	-0.104	**0.009**
SM.41:2	0.044	-0.004 (-0.006 _ -0.001)	-0.127	-0.114	**0.004**
SM.42:1	0.050	-0.006 (-0.048 _ 0.036)	-0.013	-0.011	0.777
SM.42:2	0.069	-0.017 (-0.048 _ 0.015)	-0.046	-0.041	0.291
SM.42:3	0.044	-0.002 (-0.006 _0.001)	-0.054	-0.048	0.221
SM.42:4	0.048	-0.00005 (-0.0002 _ 0.0001)	-0.023	-0.020	0.610
SM.42:6	0.019	0.00001 (-0.0001 _ 0.0001)	0.011	0.010	0.812
SM.43:1	0.087	-0.003 (-0.005_ -0.002)	-0.192	-0.171	**<0.001***
SM.43:2	0.054	-0.003 (-0.005 _ -0.001)	-0.135	-0.120	**0.002**
SM.44:2	0.047	-0.0003 (-0.001 _ 0.001)	-0.033	-0.029	0.457
SM.44:6	0.024	-0.00001 (-0.0001 _ 0.0001)	0.008	0.008	0.850
SM.	0.045	-0.0002 (-0.117 _ 0.117)	-0.0001	-0.0001	0.997

[1]Model adjusted for maternal age, gestational weight gain, parity, fish and cod liver oil intake, smoking status, education, and mode of delivery. Maternal pre-pregnancy BMI kg/m²: study exposure (independent variable), %PL: outcome variable (dependent variable). LysoPC: Lysophosphatidylcholine, PC: Phosphatidylcholine, PCaa: diacyl phosphatidylcholine, PCae: acyl-alkyl phosphatidylcholine, SM: sphingomyelin. R^2: coefficient of determination; B(%CI): unstandardized coefficient (95% confidence interval); β: standardized regression coefficient. Statistically significant p-values < 0.05 are highlighted in bold. Associations below the adjusted p-values are additionally marked by a star. Adjusted p-value = 0.05/99 = 0.0005.

Manuscript Title: The Associations of Maternal Pre-pregnancy Body Mass Index with Human Milk Fatty Acid and Phospholipid Composition in the Observational Norwegian Human Milk Study.

First Author: Talat Bashir Ahmed

Supplementary Table 8: Adjusted[1] associations of %Phospholipids in human milk samples (n = 628) with maternal pre-pregnancy body mass index (pBMI kg/m²) categories (†OW-Overweight = 25.0-29.9, ‡OB-Obese ≥ 30) with reference to normal weight group (pBMI ≤ 24.9 kg/m²).

%PL	R^2	B(95%CI)	β	Corr-Part	P-value
LysoPC14:0	0.052	-0.011 (-0.051 _ 0.029)	-0.023	-0.021	0.588
		-0.001 (-0.059 _ 0.057)	-0.001	-0.001	0.975
LysoPC 16:0	0.027	0.031 (-0.140 _ 0.201)	0.015	0.014	0.724
		0.038 (-0.208 _ 0.284)	0.014	0.012	0.764
LysoPC16:1	0.046	0.008 (0.002 _ 0.013)	0.116	0.109	**0.006**
		0.010 (0.002 _ 0.017)	0.112	0.098	**0.014**
LysoPC18:0	0.018	-0.004 (-0.037 _ 0.029)	-0.009	-0.009	0.825
		-0.001 (-0.049 _ 0.046)	-0.002	-0.002	0.960
LysoPC18:1	0.036	0.019 (-0.006 _ 0.044)	0.063	0.059	0.138
		0.035 (-0.001 _ 0.071)	0.086	0.075	0.060
LysoPC18:2	0.029	0.011 (-0.066 _ 0.088)	0.012	0.011	0.774
		0.044 (-0.067 _ 0.155)	0.036	0.031	0.434
LysoPC18:3	0.029	-0.0001 (-0.003 _ 0.003)	-0.003	-0.003	0.944
		0.001 (-0.003 _ 0.005)	0.018	0.015	0.700
LysoPC20:4	0.039	0.005 (-0.0004 _ 0.011)	0.077	0.072	0.070
		0.007 (-0.001 _ 0.016)	0.082	0.071	0.072
LysoPC22:6	0.043	0.002 (-0.004 _ 0.007)	0.022	0.021	0.603
		-0.001 (-0.009 _ 0.008)	-0.006	-0.005	0.895
LysoPC	0.026	0.060 (-0.225 _0.345)	0.018	0.017	0.678
		0.132 (-0.279 _0.542)	0.029	0.025	0.529
PCaa.30:0	0.029	-0.062 (-0.119_-0.006)	-0.092	-0.086	0.031
		-0.019 (-0.101_0.062)	-0.021	-0.019	0.641
PCaa.32:0	0.027	0.051 (-0.151 _ 0.253)	0.021	0.020	0.621
		-0.009 (-0.300 _ 0.282)	-0.003	-0.002	0.953
PCaa.32:1	0.039	0.012 (-0.010 _ 0.033)	0.044	0.042	0.292
		-0.004 (-0.035 _ 0.028)	-0.011	-0.009	0.812
PCaa.32:2	0.028	0.0001 (-0.008 _ 0.008)	0.001	0.001	0.980
		0.001 (-0.010 _ 0.012)	0.006	0.005	0.895
PCaa.32:3	0.041	0.0003 (-0.001 _ 0.002)	0.025	0.023	0.559
		-0.0001 (-0.002 _ 0.002)	-0.007	-0.006	0.872
PCaa.34:1	0.051	0.045 (-0.137 _ 0.227)	0.020	0.019	0.629
		0.095 (-0.167 _ 0.359)	0.032	0.028	0.474
PCaa.34:2	0.045	0.031 (-0.119 _ 0.181)	0.017	0.016	0.688
		-0.003 (-0.220 _ 213)	-0.001	-0.001	0.976
PCaa.34:3	0.037	-0.0004 (-0.006 _ 0.005)	-0.007	-0.007	0.862
		-0.006 (-0.013 _ 0.002)	-0.068	-0.060	0.134

PCaa.36:0	0.063	-0.005 (-0.013 _ 0.003)†	-0.050†	-0.047	0.235†
		-0.022 (-0.033 _ -0.010)‡	-0.164‡	-0.143	**<0.001***‡
PCaa.36:1	0.036	0.054 (-0.032 _ 0.140)	0.052	0.049	0.215
		0.013 (-0.110 _ 0.136)	0.009	0.008	0.835
PCaa.36:2	0.033	-0.004 (-0.454 _ 0.446)	-0.001	-0.001	0.985
		-0.191 (-0.840 _ 0.458)	-0.026	-0.023	0.563
PCaa.36:3	0.038	-0.023 (-0.085 _ 0.039)	-0.031	-0.029	0.466
		-0.044 (-0.134_ 0.045)	-0.044	-0.039	0.329
PCaa.36:4	0.047	0.001 (-0.015 _ 0.017)	0.005	0.005	0.902
		-0.001 (-0.024 _ 0.022)	-0.004	-0.003	0.933
PCaa.36:5	0.092	-0.0001(-0.002 _ 0.002)	-0.006	-0.006	0.886
		-0.003 (-0.006 _ 0.001)	-0.068	-0.059	0.125
PCaa.38:3	0.051	-0.004 (-0.033 _ 0.025)	-0.012	-0.011	0.777
		-0.013 (-0.055_ 0.028)	-0.029	-0.025	0.527
PCaa.38:4	0.064	0.008 (-0.019 _ 0.036)	0.025	0.024	0.543
		-0.0003 (-0.040 _ 0.039)	-0.001	-0.001	0.985
PCaa.38:5	0.128	-0.003 (-0.010 _0.004)	-0.031	-0.029	0.435
		-0.010 (-0.020 _ -0.0003)	-0.088	-0.077	0.043
PCaa.38:6	0.125	-0.004 (-0.009 _ 0.001)	-0.058	-0.055	0.150
		-0.006 (-0.014 _ 0.002)	-0.065	-0.057	0.135
PCaa.40:4	0.053	-0.001 (-0.002 _ 0.001)	-0.033	-0.032	0.431
		-0.001 (-0.003 _ 0.002)	-0.020	-0.018	0.655
PCaa.40:5	0.038	-0.001 (-0.004 _ 0.002)	-0.022	-0.020	0.607
		-0.003 (-0.007 _ 0.002)	-0.050	-0.043	0.275
PCaa.40:6	0.140	-0.002 (-0.011 _ 0.007)	-0.015	-0.014	0.705
		-0.013 (-0.026 _ 0.000006)	-0.084	-0.074	0.050
PCae.30:0	0.027	-0.002 (-0.007 _ 0.002)	-0.047	-0.044	0.268
		-0.003 (-0.010 _0.003)	-0.051	-0.044	0.267
PCae.32:0	0.022	-0.007 (-0.022 _ 0.008)	-0.036	-0.034	0.393
		-0.010 (-0.032 _ 0.012)	-0.041	-0.036	0.373
PCae.32:1	0.056	0.005 (-0.001 _ 0.010)	0.070	0.065	0.097
		0.0002 (-0.008 _ 0.008)	0.003	0.002	0.953
PCae.34:0	0.075	-0.010 (-0.025 _ 0.005)	-0.054	-0.051	0.191
		-0.030 (-0.052 _ -0.009)	-0.123	-0.108	0.006
PCae.34:1	0.036	-0.003 (-0.014 _ 0.009)	-0.020	-0.019	0.634
		-0.009 (-0.026 _ 0.007)	-0.052	-0.045	0.255
PCae.34:2	0.064	-0.010 (-0.017 _ -0.002)	-0.103	-0.097	0.013
		-0.014 (-0.025 _ -0.003)	-0.108	-0.094	0.016
PCae.34:3	0.062	-0.004 (-0.012 _ 0.004)	-0.044	-0.041	0.295
		-0.013 (-0.024 _ -0.002)	-0.107	-0.093	0.017
PCae.36:2	0.049	-0.008 (-0.019 _ 0.003)†	-0.061†	-0.057	0.148†
		-0.025 (-0.040 _ -0.009)‡	-0.142‡	-0.124	**0.002**‡
PCae.36:3	0.075	-0.009 (-0.016 _ -0.002)†	-0.101†	-0.095	**0.015**†
		-0.019 (-0.030 _ -0.009)‡	-0.160‡	-0.140	**<0.001**‡
PCae.36:4	0.041	0.0002 (-0.004 _ 0.005)	0.004	0.004	0.920
		-0.005 (-0.012 _ 0.001)	-0.072	-0.063	0.111
PCae.36:5	0.046	0.002 (-0.001 _ 0.005)	0.048	0.045	0.259
		-0.002 (-0.007 _ 0.003)	-0.041	-0.036	0.361
PCae.38:3	0.032	-0.002 (-0.005 _ 0.001)	-0.068	-0.064	0.110
		-0.006 (-0.010 _ -0.002)	-0.141	-0.123	0.002
PC	0.047	0.045 (-0.992 _ 1.082)	0.004	0.003	0.932
		-0.376 (-1.872 _ 1.119)	-0.022	-0.019	0.621

SM.33:1	0.042	-0.007 (-0.017 _ 0.002)	-0.063	-0.059	0.135
		-0.012 (-0.025 _ 0.002)	-0.076	-0.067	0.093
SM.34:1	0.031	0.033 (-0.145_ 0.211)	0.015	0.014	0.718
		-0.049 (-0.305 _ 0.208)	-0.017	-0.015	0.709
SM.34:2	0.036	0.007 (-0.007 _ 0.020)	0.040	0.038	0.344
		0.0002 (-0.019 _ 0.020)	0.001	0.001	0.983
SM.35:1	0.044	-0.005 (-0.021 _ 0.011)	-0.027	-0.026	0.516
		-0.020 (-0.042 _ 0.003)	-0.078	-0.068	0.087
SM.36:1	0.089	0.264 (0.002 _ 0.527)	0.081	0.076	0.048
		0.300 (-0.079 n_ 0.679)	0.069	0.060	0.120
SM.36:2	0.056	0.025 (-0.003 _ 0.052)	0.074	0.071	0.077
		0.001 (-0.038 _ 0.041)	0.003	0.003	0.948
SM.37:1	0.046	0.004 (-0.014 _ 0.022)	0.019	0.018	0.653
		-0.014 (-0.040 _ 0.012)	-0.049	-0.042	0.284
SM.38:1	0.029	-0.062 (-0.175 _ 0.052)	-0.045	-0.042	0.288
		0.033 (-0.132 _ 0.197)	0.018	0.016	0.696
SM.38:2	0.025	0.008 (-0.009 _ 0.025)	0.040	0.037	0.348
		0.010 (-0.015_ 0.034)	0.035	0.030	0.446
SM.39:1	0.066	-0.010 (-0.032 _ 0.012)	-0.037	-0.035	0.369
		-0.017 (-0.049 _ 0.014)	-0.048	-0.042	0.286
SM.39:2	0.021	-0.002 (-0.005 _ 0.002)	-0.035	-0.033	0.407
		-0.004 (-0.010 _ 0.001)	-0.070	-0.061	0.129
SM.40:1	0.043	0.147 (-0.958 _ 1.253)	0.011	0.010	0.794
		0.384 (-1.211 _1.980)	0.021	0.019	0.636
SM.40:2	0.036	-0.007 (-0.055 _ 0.041)	-0.012	-0.011	0.785
		-0.030 (-0.099 _ 0.040)	-0.038	-0.033	0.399
SM.40:3	0.038	-0.002 (-0.007 _ 0.003)	-0.035	-0.033	0.409
		-0.008 (-0.015 _ -0.001)	-0.099	-0.086	0.030
SM.41:1	0.040	-0.092 (-0.,183 _ -0.002)	-0.084	-0.079	0.046
		-0.100 (-0.231 _ 0.031)	-0.068	-0.059	0.135
SM.41:2	0.036	-0.009 (-0.035 _ 0.016)	-0.031	-0.029	0.469
		-0.033 (-0.069 _ 0.004)	-0.080	-0.070	0.079
SM.42:1	0.054	-0.312 (-0.717 _ 0.094)	-0.063	-0.059	0.132
		0.077 (-0.508 _ 0.662)	0.012	0.010	0.795
SM.42:2	0.068	-0.039 (-0.342_ 0.264)	-0.011	-0.010	0.800
		-0.175 (-0.612 _ 0.263)	-0.035	-0.031	0.433
SM.42:3	0.045	-0.011 (-0.044 _ 0.022)	-0.027	-0.025	0.525
		-0.030 (-0.078 _ 0.018)	-0.056	-0.049	0.218
SM.42:4	0.050	-0.0003 (-0.002 _ 0.002)	-0.013	-0.012	0.764
		-0.002 (-0.005 _ 0.001)	-0.049	-0.043	0.275
SM.42:6	0.022	-0.001 (-0.002 _ 0.001)	-0.034	-0.032	0.424
		0.001 (-0.001 _ 0.002)	0.035	0.031	0.440
SM.43:1	0.080	-0.023 (-0.037 _ -0.009)	-0.131	-0.123	**0.002†**
		-0.031 (-0.051 _ -0.010)	-0.130	-0.113	**0.004‡**
SM.43:2	0.050	-0.009 (-0.026 _ 0.008)	-0.043	-0.040	0.311
		-0.033 (-0.058 _ -0.008)	-0.119	-0.104	0.009
SM.44:2	0.048	-0.001 (-0.009 _ 0.007)	-0.011	-0.010	0.801
		-0.007 (-0.019 _ 0.004)	-0.057	-0.050	0.209
SM.44:6	0.036	-0.002 (-0.003 _ -0.00006)	-0.087	-0.081	0.041
		0.001 (-0.001 _ 0.003)	0.053	0.046	0.246
SM	0.045	-0.105 (-1.232 _ 1.022)	-0.008	-0.007	0.855
		0.245 (-1.381_ 1,871)	0.013	0.012	0.768

BMI-categories (OW† Overweight =25.0-29.9, OB‡ obese≥30 kg/m²). [1]Model adjusted for maternal age, gestational weight gain, parity, fish and cod liver oil intake, smoking, education, and mode of delivery. Maternal pre-pregnancy BMI kg/m²: study exposure (independent variable), %PL: percentage phospholipid composition as

outcome variable (dependent variable). LysoPC: Lysophosphatidylcholine, PC: Phosphatidylcholine, PCaa: diacyl phosphatidylcholine, PCae: acyl-alkyl phosphatidylcholine, SM: sphingomyelin. R^2: coefficient of determination, B(%CI): unstandardized coefficient (95% confidence interval), β: standardized regression coefficient. Statistically significant p-values < 0.05 are highlighted in bold. Associations below the adjusted p-values are additionally marked by a star. Adjusted p-value = 0.05/99 = 0.0005**.**

Manuscript Title: The Associations of Maternal Pre-pregnancy Body Mass Index with Human Milk Fatty Acid and Phospholipid Composition in the Observational Norwegian Human Milk Study.

First Author: Talat Bashir Ahmed

Supplementary Table 9: Adjusted associations with p-values <0.05† of %lipid metabolite in human milk samples (n=628) with the study covariates.

Study Covariates	%Lipid metabolites	B (95% CI)	β	P-value
[1] Cod liver oil consumption (servings in days per year)	n3-PUFA	0.001 (0.001 _ 0.001)	0.259	<0.001*
	LCPUFA	0.001 (0.0004 _ 0.001)	0.299	<0.001*
	PCaa.36:5	0.00001 (0.00001_0.00002)	0.223	<0.001*
	PCaa.38:5	0.00006 (0.00004 _ 0.00008)	0.267	<0.001*
	PCaa.38:6	0.00004 (0.00002 _ 0.00005)	0.230	<0.001*
	PCaa.40:6	0.00008 (0.00005 _ 0.0001)	0.264	<0.001*
[2] Total meals of fatty fish dinner (servings in days per year)	n3-PUFA	0.003 (0.002 _ 0.005)	0.181	<0.001*
	LCPUFA	0.002 (0.001_ 0.003)	0.186	<0.001*
[3] Gestational weight gain	MCSFA	-0.089 (-0.137 _-0.041)	-0.162	<0.001*
	MUFA	0.093 (0.052 _ 0.134)	0.193	<0.001*
	C16:1n-7	0.032 (0.023 _0.040)	0.297	<0.001*
	C18:1n-7	0.010 (0.006 _0.013)	0.252	<0.001*
	SM.36:1	0.054 (0.032 _ 0.075)	0.214	<0.001*
	SM.36:2	0.004 (0.002 _ 0.006)	0.154	<0.001
[4] Mode of delivery (cesarean delivery)	PCaa.38:3	0.066 (0.030 _ 0.101)	0.153	<0.001
	PCaa.38:4	0.037 (0.004 _ 0.071)	0.091	0.030
	PCaa.40:4	0.003 (0.001 _ 0.005)	0.121	0.004
[5] Parity (multiparity)	PCaa.40:6	-0.014 (-0.022 _ -0-006)	-0.134	<0.001*
	PCae.32:1	-0.014 (-0.020 _ -0.009)	-0.235	<0.001*
	PCae.34:3	-0.012 (-0.019 _ -0.004)	-0.139	0.002
	SM.42:2	-0.530 (-0.830 _ -0.229)	-0.155	<0.001
[6] Maternal Smoking	C22:6n-3	-0.051 (-0.079 _ -0.023)	-0.119	<0.001*
[7] Maternal Age	C20:5n-3	0.003 (0.001 _ 0.004)	0.151	<0.001*
	C22:6n-3	0.007 (0.003 _ 0.011)	0.133	<0.001*

†Associations included with p-values < 0.05 and those less than adjusted p-values are additionally marked by a star. Bonferroni Adjusted p-value = 0.05/99 = 0.0005. [1] Defined as total servings in days of cod liver oil in the previous year. Model adjusted for maternal age, education, body mass index, parity, maternal smoking, vegetarian dinner, fatty fish dinner, and gestational weight gain. [2] Defined as total meals of fatty fish dinner or bread meals

in days (where each bread meal counted as 1/7 dinner size) per year. Model adjusted for maternal age, gestational weight gain, pre-pregnancy BMI, education, parity, smoking, cod liver oil consumption and vegetarian dinner. [3]Defined as the difference in weight gain in kg between maternal weight at delivery to pre-pregnancy maternal weight. Model adjusted for maternal age, gestational age, fish and cod liver oil consumption, mode of delivery, education, body mass index, smoking, and parity. [4] Defined as vaginal or cesarean delivery. Model adjusted for maternal age, body mass index, gestational weight gain, gestational age, fish and cod liver oil consumption, education, parity, and smoking status. [5] Defined as primiparous vs. multiparous. Model adjusted for maternal age, body mass index, gestational weight gain, fish and cod liver oil consumption, education, and smoking. [6] Defined as never smoke vs. current+ past smokers. Model adjusted for maternal age, fish and cod liver oil consumption, education, body mass index, and gestational weight gain. [7]Maternal age in years, model adjusted for pre-pregnancy BMI, gestational weight gain, fish and cod liver oil consumption, parity, mode of delivery, education, and smoking status.

Manuscript Title: The Associations of Maternal Pre-pregnancy Body Mass Index with Human Milk Fatty Acid and Phospholipid Composition in the Observational Norwegian Human Milk Study.

First Author: Talat Bashir Ahmed

Supplementary Table 10: Adjusted[1] associations of %Fatty acids in human milk samples (n = 650 including outliers) with maternal pre-pregnancy body mass index (pBMI kg/m²).

%FA	R^2	B (95% CI)	β	Corr-Part	P-value
C8:0	0.044	-0.002 (-0.004 _ -0.003)	-0.102	-0.091	0.019
C10:0	0.054	-0.005 (-0.011_ 0.002)	-0.057	-0.051	0.189
C12:0	0.060	-0.012 (-0.042 _ 0.019)	-0.032	-0.028	0.466
C13:0	0.056	-0.002 (-0.003_ -0.001)	-0.175	-0.156	**<0.001**
C14:0	0.067	-0.012 (-0.039 _ 0-014)	-0.040	-0.035	0.359
C15:0	0.105	-0.004 (-0.005 _ -0.002)	-0.186	-0.166	**<0.001**
C16:0	0.036	0.015 (-0.017 _ 0.046)	0.039	0.035	0.371
C17:0	0.054	-0.001 (-0.002 _ -0.0001)	-0.103	-0.092	0.018
C18:0	0.043	-0.035 (-0.059 _ -0.011)	-0.124	-0.111	**0.005**
C22:0	0.051	-0.001 (-0.002_ -0.0002)	-0.107	0.095	**0.014**
C14:1	0.036	0.0003 (-0.001 _ 0.002)	0.026	0.023	0.556
C15:1	0.058	-0.0003 (-0.001 _ 0.00006)	-0.070	-0.062	0.108
C16:1t	0.040	0.0001 (-0.0003 _ 0.001)	0.024	0.021	0.587
C18:1t	0.038	-0.002 (-0.006 _ 0.001)	-0.057	-0.051	0.192
C22:1t	0.129	-0.001 (-0.001 _ -0.00009)	-0.095	-0.084	0.023
C16:1n-7	0.142	0.027 (0.016 _ 0.039)	0.197	0.175	**<0.001***
C18:1n-7	0.116	0.009 (0.005 _ 0.013)	0.182	0.162	**<0.001***
C18:1n-9	0.071	0.088 (0.041 _ 0.135**)**	0.158	0.141	**<0.001***
C20:1n-9	0.134	-0.003 (-0.005 _ -0.001)	-0.118	-0.105	0.005
C18:2n-6	0.033	-0.046 (-0.095 _ 0.002)	-0.083	-0.073	0.060
C18:3n-6	0.042	0.0004 (-0.0003_ 0.001)	0.054	0.048	0.215
C18:3n-3	0.038	-0.004 (-0.01_ 0.002)	-0.062	-0.056	0.157
C20:2n-6	0.031	-0.002 (-0.005 _ 0.001)	-0.061	-0.054	0.165
C20:3n-9	0.049	-0.00005 (-0.0001 _ 0.00006)	-0.039	-0.035	0.371
C20:3n-6	0.063	0.001 (-0.0004 _ 0.003)	0.059	0.053	0.169
C20:3n-3	0.047	-0.0002 (-0.0004 _0.000004)	-0.084	-0.075	0.054
C20:4n-6	0.052	0.001 (-0.001 _ 0.002)	0.037	0.033	0.400
C.20:5n-3	0.330	-0.002 (-0.003_ -0.001)	-0.135	-0.120	**<0.001***
C.22:4n-6	0.067	0.001 (0.0002 _ 0.001)	0.119	0.106	**0.006**
C22:5n-6	0.020	-0.0002 (-0.0005_0.00006)	-0.066	-0.060	0.132
C22:5n-3	0.251	-0.002 (-0.002 _ -0.001)	-0.119	-0.106	**0.002**
C22:6n-3	0.320	-0.007 (-0.010 _ -0.003)	-0.139	-0.124	**<0.001***
SFA	0.065	-0.058 (-0.126 _ 0.009)	-0.073	-0.065	0.090
MCSFA	0.064	-0.030 (-0.092 _0.031)	-0.042	-0.037	0.332
ODCFA	0.100	-0.007 (-0.009 _ -0.004)	-0.204	-0.182	**<0.001**

MUFA	0.098	0.122 (0.069 _ 0.175)	0.191	0.170	<**0.001***
tFA	0.036	-0.003 (-0.007 _ 0.001)	-0.067	-0.059	0.128
PUFA	0.033	-0.061 (-0.115 _-0.006)	-0.096	-0.08	0.029
N6-PUFA	0.032	-0.046 (-0.096 _ 0.004)	-0.079	-0.070	0.074
N3-PUFA	0.216	-0.015 (-0.023 _ -0.006)	-0.136	-0.135	<**0.001***
N6/N3-PUFA	0.248	0.034 (0.002 _ 0.066)	0.081	0.072	0.037
LCPUFA	0.195	-0.009 (-0.015 _ -0.002)	-0.104	-0.092	0.010
N6-LCPUFA	0.056	0.002 (-0.001 _ 0.005)	0.065	0.058	0.131
N3-LCPUFA	0.321	-0.011 (-0.016 _ -0.005)	-0.140	-0.125	<**0.001***
N6/N3-LCPUFA	0.400	0.021 (0.012_ 0.029)	0.172	0.153	<**0.001***
Total FA	0.061	0.293 (0.129 _0.457)	0.152	0.135	<**0.001**

[1]Model adjusted for maternal age, gestational weight gain, parity, fish and cod liver oil intake, smoking, education, and mode of delivery. Maternal pre-pregnancy BMI kg/m^2: study exposure (independent variable), %FA: outcome variable (dependent variable). R^2: coefficient of determination; B(%CI): unstandardized coefficient (95% confidence interval); β: standardized regression coefficient. Statistically significant p-values < 0.05 are highlighted in bold. Associations below the adjusted p-values are additionally marked by a star. Adjusted p-value = 0.05/99 = 0.0005.

Manuscript Title: The Associations of Maternal Pre-pregnancy Body Mass Index with Human Milk Fatty Acid and Phospholipid Composition in the Observational Norwegian Human Milk Study.

First Author: Talat Bashir Ahmed

Supplementary Table 11: Adjusted[1] associations of %Phospholipid species in human milk samples (n = 650, including outliers) with maternal pre-pregnancy body mass index (pBMI, kg/m²).

%PL	R^2	B (95% CI)	β	Corr-Part	P-value
LysoPC14:0	0.050	0.002 (-0.002 _ 0.007)	0.047	0.042	0.282
LysoPC16:0	0.026	0.008 (-0.01_ 0.027)	0.039	0.035	0.377
LysoPC16:1	0.045	0.001 (0.001 _ 0.002)	0.169	0.150	**<0.001***
LysoPC18:0	0.019	-0.002 (-0.006 _ 0.001)	-0.059	-0.052	0.185
LysoPC18:1	0.047	0.005 (0.003 _ 0.008)	0.170	0.151	**<0.001***
LysoPC18:2	0.031	0.006 (-0.003 _ 0.014)	0.055	0.049	0.211
LysoPC18:3	0.028	0.0001 (-0.0001 _ 0.0004)	0.054	0.049	0.216
LysoPC20:4	0.034	0.001 (0.0001 _ 0.001)	0.107	0.095	**0.015**
LysoPC22:6	0.043	0.0002 (-0.0003_ 0.001)	0.031	0.028	0.474
LysoPC	0.026	0.022 (-0.010 _0.053)	0.060	0.053	0.175
PCaa.30:0	0.025	-0.002 (-0.008 _ 0.004)	-0.028	-0.025	0.520
PCaa.32:0	0.024	0.005 (-0.016 _ 0.026)	0.021	0.019	0.636
PCaa.32:1	0.037	0.001 (-0.001 _ 0.003)	0.040	0.035	0.364
PCaa.32:2	0.031	-0.00006 (-0.001 _ 0.001)	-0.007	-0.006	0.877
PCaa.32:3	0.046	-0.00001(-0.0001_ 0.0001)	-0.007	-0.007	0.865
PCaa.34:1	0.051	0.013 (-0.005 _ 0.032)	0.061	0.054	0.162
PCaa.34:2	0.044	0.003 (-0.013 _ 0.018)	0.016	0.014	0.720
PCaa.34:3	0.041	-0.0003 (-0.001_ 0.0002)	-0.052	-0.046	0.238
PCaa.36:0	0.048	-0.001 (-0.002 _ -0.001)	-0.147	-0.139	**<0.001***
PCaa.36:1	0.033	0.004 (-0.005 _ 0.012)	0.036	0.032	0.416
PCaa.36:2	0.035	-0.015 (-0.061 _ 0.032)	-0.027	-0.024	0.536
PCaa.36:3	0.047	-0.002 (-0.008 _ 0.005)	-0.025	-0.022	0.570
PCaa.36:4	0.047	-0.00004 (-0.002 _0.002)	-0.002	-0.002	0.962
PCaa.36:5	0.112	-0.0001(-0.0004 _ 0.00005)	-0.065	-0.057	0.125
PCaa.38:3	0.059	-0.001 (-0.004 _ 0.002)	-0.037	-0.033	0.395
PCaa.38:4	0.065	0.0003 (-0.003 _ 0.003)	0.009	0.008	0.836
PCaa.38:5	0.142	-0.001 (-0.002 _ -0.0001)	-0.096	-0.085	**0.020**
PCaa.38:6	0.120	-0.001 (-0.001 _0.00002)	-0.079	-0.070	0.061
PCaa.40:4	0.058	-0.0001 (-0.0002 _ 0.00007)	-0.049	-0.044	0.258
PCaa.40:5	0.053	-0.0002 (-0.001 _ 0.00009)	-0.062	-0.056	0.150
PCaa.40:6	0.144	-0.001 (-0.002 _ -0.00004)	-0.085	-0.075	**0.041**
PCae.30:0	0.021	-0.00009 (-0.001 _ 0.0003)	-0.018	-0.016	0.680
PCae.32:0	0.019	-0.001 (-0.002 _ 0.001)	-0.040	-0.035	0.370
PCae.32:1	0.047	0.00007 (-0.001 _ 0.001)	0.011	0.010	0.793

PCae.34:0	0.073	-0.002 (-0.004 _ -0.001)	-0.130	-0.116	**0.002**
PCae.34:1	0.031	-0.0003 (-0.001 _ 0.001)	-0.022	-0.019	0.621
PCae.34:2	0.055	-0.001 (-0.002_ -0.0002)	-0.112	-0.100	**0.010**
PCae.34:3	0.069	-0.001 (-0.002 _ -0.0001)	-0.096	-0.085	**0.027**
PCae.36:2	0.044	-0.001 (-0.002 _ -0.0002)	-0.104	-0.093	**0.017**
PCae.36:3	0.060	-0.001 (-0.002 _ -0.001)	-0.141	-0.125	**0.001**
PCae.36:4	0.037	-0.0003 (-0.001 _ 0.0001)	-0.056	-0.050	0.198
PCae.36:5	0.039	-0.00004 (-0.0004_ 0.0003)	-0.010	-0.009	0.826
PCae.38:3	0.031	-0.0004 (-0.001 _ -0.0001)	-0.138	-0.123	**0.002**
PC	0.048	-0.007 (-0.114_ 0.100)	-0.006	-0.005	0.894
SM.33:1	0.029	-0.001 (-0.003 _ -0.0003)	-0.117	-0.104	**0.008**
SM.34:1	0.027	-0.011 (-0.030_ 0.008)	-0.049	-0.044	0.264
SM.34:2	0.030	-0.00004 (-0.001 _0.002)	0.003	0.002	0.951
SM.35:1	0.050	-0.002 (-0.004 _-0.001)	-0.116	-0.104	**0.008**
SM.36:1	0.089	0.018 (-0.010_ 0.046)	0.053	0.047	0.218
SM.36:2	0.056	0.0001 (-0.003 _ 0.003)	0.004	0.004	0.925
SM.37:1	0.052	-0.001 (-0.003 _ 0.001)	-0.058	-0.052	0.179
SM.38:1	0.028	-0.004 (-0.016 _ 0.008)	-0.030	-0.027	0.491
SM.38:2	0.024	0.001 (-0.001 _ 0.003)	0.048	0.043	0.273
SM.39:1	0.077	-0.003 (-0.005_-0.001)	-0.112	-0.099	**0.009**
SM.39:2	0.030	-0.0004 (-0.001 _ -0.00004)	-0.096	-0.085	**0.030**
SM.40:1	0.043	0.036 (-0.077_0.149)	0.027	0.024	0.533
SM.40:2	0.040	-0.003 (-0.007 _ 0.002)	-0.044	-0.039	0.315
SM.40:3	0.036	-0.0003 (-0.001 _ 0.0001)	-0.054	-0.048	0.219
SM.41:1	0.045	-0.013 (-0.022 _ -0.003)	-0.114	0.101	**0.009**
SM.41:2	0.047	-0.004 (-0.007 _ -0.002)	-0.139	0.123	**0.002**
SM.42:1	0.047	0.0001 (-0.042 _ 0.042)	0.0002	0.0002	0.995
SM.42:2	0.067	-0.017 (-0.049_ 0.014)	-0.046	0.041	0.283
SM.42:3	0.032	-0.002 (-0.006 _ 0.001)	-0.050	0.044	0.256
SM.42:4	0.040	-0.00002 (-0.0002_ 0.0001)	-0.011	0.010	0.797
SM.42:6	0.015	0.00001 (-0.0001_ 0.0001)	0.007	0.006	0.871
SM.43:1	0.081	-0.003 (-0.005_ -0.002)	-0.182	0.162	**<0.001***
SM.43:2	0.056	-0.003 (-0.005 _ -0.001)	-0.142	-0.126	**0.001**
SM.44:2	0.049	-0.0003 (-0.001 _ 0.001)	-0.033	-0.030	0.442
SM.44:6	0.026	0.00002 (-0.0001 _ 0.0001)	0.011	0.010	0.799
SM	0.044	-0.014 (-0.131_ 0.102)	-0.011	-0.009	0.809

[1]Model adjusted for maternal age, gestational weight gain, parity, fish and cod liver oil intake, smoking status, education, and mode of delivery. Maternal pre-pregnancy BMI kg/m²: study exposure (independent variable), %PL: percentage phospholipid composition as outcome variable (dependent variable). LysoPC: Lysophosphatidylcholine, PC: Phosphatidylcholine, PCaa: diacyl phosphatidylcholine, PCae: acyl-alkyl phosphatidylcholine, SM: sphingomyelin. R^2: coefficient of determination; B(%CI): unstandardized coefficient (95% confidence interval); β: standardized regression coefficient. Statistically significant p-values < 0.05 are highlighted in bold. Associations below the adjusted p-values are additionally marked by a star. Adjusted p-value = 0.05/99 = 0.0005.

Manuscript Title: The Associations of Maternal Pre-pregnancy Body Mass Index with Human Milk Fatty Acid and Phospholipid Composition in the Observational Norwegian Human Milk Study.

First Author: Talat Bashir Ahmed

Supplementary Table 12: Undjusted[1] associations of centered log-ratio transformed Phospholipid species in human milk samples (n = 562) with maternal pre-pregnancy body mass index (pBMI).

Clr-Phospholipid species (clr-PL)	B (95% CI)	β	P-value
LysoPC.16:1	0.013 (0.004 _ 0.022)	0.117	0.005
LysoPC.18:1	0.011 (0.005_ 0.017)	0.144	<0.001*
LysoPC.20:4	0.010 (0.001 _ 0.019)	0.091	0.030
PCaa.36:0	-0.006 (-0.012 _-0.0005)	-0.089	0.034
PCaa.38:5	-0.013 (-0.022 _ -0.003)	-0.110	0.009
PCaa.38:6	-0.014 (-0.027 _ -0.001)	-0.089	0.036
PCaa.40:6	-0.012 (-0.022_ -0.002)	-0.101	0.017
PCae.36:0	-0.007 (-0.013 _-0.001)	-0.091	0.031
PCae.38:2	-0.006 (-0.012 _ -0.001)	-0.091	0.032
PCae.38:3	-0.008 (-0.015 _ -0.002)	-0.107	0.011
PCae.38:5	0.010 (0.003_ 0.018)	0.109	0.010
SM.36:1	0.006 (0.0005 _ 0.011)	0.090	0.032

LysoPC: Lysophosphatidylcholine, PC: Phosphatidylcholine, PCaa: diacyl phosphatidylcholine, PCae: acyl-alkyl phosphatidylcholine, SM: sphingomyelin. B(%CI): unstandardized coefficient (95% confidence interval); β: standardized regression coefficient. Statistically significant (p-values < 0.05) associations are included. Associations below the adjusted p-values are additionally marked by a star. Adjusted p-value = 0.05/76 = 0.0006.

Manuscript Title: The Associations of Maternal Pre-pregnancy Body Mass Index with Human Milk Fatty Acid and Phospholipid Composition in the Observational Norwegian Human Milk Study.

First Author: Talat Bashir Ahmed

Supplementary Table 13: Adjusted[1] associations of centered log-ratio transformed Phospholipid species in human milk samples (n = 562) with maternal pre-pregnancy body mass index (pBMI).

Clr-Phospholipid species (clr-PL)	B (95% CI)	β	P-value
LysoPC.16:1	0.015 (0.004 _ 0.025)	0.135	0.006
LysoPC.18:1	0.011 (0.004_ 0.018)	0.146	0.002
LysoPC.20:4	0.017 (0.007 _ 0.027)	0.157	0.001
PCaa.36:0	-0.007 (-0.013 _ -0.001)	-0.099	0.026
PCaa.38:5	-0.011 (-0.021 _ -0.001)	-0.093	0.031
PCaa.38:6	-0.014 (-0.001 _ -0.089)	-0.090	0.042
PCaa.40:6	-0.011 (-0.020_ -0.001)	-0.091	0.026
PCae.36:0	-0.006 (-0.012 _0.000)	-0.085	0.045
PCae.38:2	-0.007 (-0.012 _ -0.001)	-0.099	0.019
PCae.38:3	-0.008 (-0.015 _ -0.002)	-0.107	0.011
PCae.38:5	0.010 (0.003_ 0.018)	0.109	0.010
SM.36:1	0.008 (0.003 _ 0.013)	0.127	0.003

[1]Model adjusted for maternal age, gestational weight gain, parity, fish and cod liver oil consumption, smoking, education, and mode of delivery. B(%CI): unstandardized coefficient (95% confidence interval); β: standardized regression coefficient. Associations included with p-value < 0.05. Adjusted p-value = 0.05/76 = 0.0006.
